# Regulation of cardiac ferroptosis in diabetic human heart failure: uncovering molecular pathways and key targets

**DOI:** 10.1038/s41420-024-02044-w

**Published:** 2024-06-01

**Authors:** Flobater I. Gawargi, Paras K. Mishra

**Affiliations:** Department of Cellular and Integrative Physiology, University of Nebraska Medical Center, Omaha, NE USA

**Keywords:** Cardiovascular diseases, Cardiovascular biology

## Abstract

Diabetes significantly increases the risk of heart failure by inducing myocardial cell death, potentially through ferroptosis—an iron-dependent, non-apoptotic cell death pathway characterized by lipid peroxidation. The role of cardiac ferroptosis in human heart failure, however, remains poorly understood. In this study, we compared cardiac ferroptosis in humans with diabetic heart failure to that in healthy controls. Our findings reveal that diabetes not only intensifies myocardial cell death but also upregulates markers of ferroptosis in human hearts. This is linked to decreased transcription and activity of glutathione peroxidase-4 (GPX4), influenced by reduced levels of activating transcription factor-4 (ATF4) and nuclear factor erythroid-2-related factor-2 (NRF2), and downregulation of glutathione reductase (GSR). Additionally, diabetic hearts showed an increased labile iron pool due to enhanced heme metabolism by heme oxygenase-1 (HMOX1), elevated iron import via divalent metal transporter-1 (DMT1), reduced iron storage through ferritin light chain (FLC), and decreased iron export via ferroportin-1 (FPN1). The reduction in FPN1 levels likely results from decreased stabilization by amyloid precursor protein (APP) and diminished NRF2-mediated transcription. Furthermore, diabetes upregulates lysophosphatidylcholine acyltransferase-3 (LPCAT3), facilitating the integration of polyunsaturated fatty acids (PUFA) into phospholipid membranes, and downregulates acyl-CoA thioesterase-1 (ACOT1), which further promotes ferroptosis. LC–MS/MS analysis identified several novel proteins implicated in diabetes-induced cardiac ferroptosis, including upregulated ceruloplasmin, which enhances iron metabolism, and cytochrome b-245 heavy chain (CYBB), a key component of NADPH oxidase that aids in the production of reactive oxygen species (ROS), along with downregulated voltage-dependent anion-selective channel protein-2 (VDAC2), essential for maintaining mitochondrial membrane potential. In conclusion, our study not only confirms the presence and potentially predominant role of cardiac ferroptosis in humans with diabetic heart failure but also elucidates its molecular mechanisms, offering potential therapeutic targets to mitigate heart failure complications in diabetic patients.

## Introduction

Diabetes mellitus, a highly prevalent disease, significantly elevates the risk of heart failure [1]. Despite hyperglycemia being a hallmark of diabetes, intensive glycemic control in diabetic patients has not proven effect in reducing the risk of heart failure [2, 3]. Recent studies indicate that ferroptosis, an iron-dependent form of non-apoptotic cell death, plays a key role in cardiovascular diseases, and inhibiting this process may protect against cardiomyopathy [4, 5]. However, diabetic hearts exhibit distinct features that set them apart from non-diabetic hearts [6].

The DMCM-AHEAD (Diabetes and Lipid Accumulation and Heart Transplant) trial, focusing on patients with type 2 diabetes mellitus (T2DM), identified intramyocardial lipid accumulation as a unique characteristic of diabetic heart failure, which is absent in non-diabetic heart failure patients [7]. Moreover, elevated iron stores have been specifically associated with T2DM in women [8]. Additionally, there is an increased presence of mitochondrial reactive oxygen species (ROS) in the hearts of diabetic individuals [9]. These factors collectively suggest that ferroptosis could be central to the pathogenesis of diabetic heart failure [10, 11].

Considering that apoptosis contributes minimally to human heart failure, with only 80–250 cardiomyocytes per 100,000 affected, non-apoptotic cell death mechanisms, including ferroptosis, gain prominence in this context [12, 13]. Given the limited regenerative capacity of the adult human heart and the irreversible loss of cardiomyocytes, it is crucial to explore myocardial cell death mechanisms to prevent and treat heart failure in diabetic patients [14, 15]. The combined presence of excess lipids and iron strongly supports a pivotal role for ferroptosis in myocardial cell death among diabetic patients [10].

Despite the critical role of ferroptosis in myocardial cell death, particularly in diabetes, reports on human cardiac ferroptosis are lacking, highlighting the need for comprehensive studies [10, 16]. This study aims to address this gap by examining heart tissue samples from both healthy subjects and diabetic patients, matched for age, sex and race, following established guidelines for evaluating myocardial cell death [15]. Our findings demonstrate that diabetes induces myocardial ferroptosis, contributing significantly to cell death in human heart failure, and underscore the importance of further research into this cell death pathway.

## Results

### Comparative cohort analysis of type 2 diabetes mellitus (T2DM) patients with heart failure and healthy subjects

The diabetic human hearts manifest considerable molecular variability within the same cohort, likely influenced by factors such as age, sex, race, and etiology [17]. To minimize this variability, we carefully curated heart tissue samples from T2DM heart failure patients and healthy subjects, ensuring a close match in terms of age, sex, and race as detailed in Table 1. These de-identified heart tissue samples were obtained from the BioIVT company.

**Table 1:** Demographic information of healthy control subjects and diabetic heart failure patients.

Sample type	Healthy control subjects	Type 2 diabetic heart failure patients
Age	65 ± 2.1	71 ± 0.0
Diagnosis	None	Interstitial fibrosis, interstitial pericarditis
Sex	Male	Male
Ethnicity	White	White

### Enhanced myocardial cell death in heart failure patients with diabetes

**Fig. 1:**
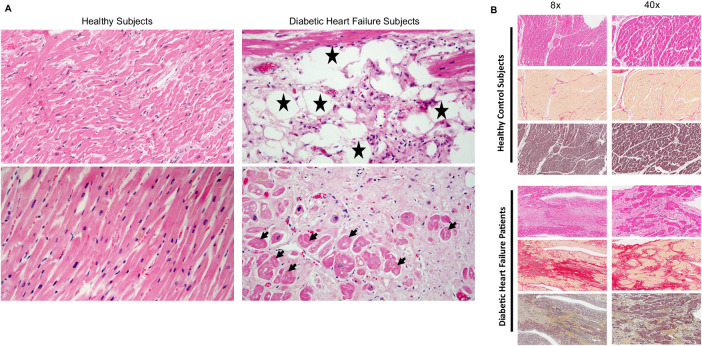
Diabetes increases cell death in human heart failure. **A** Histological sections of the heart from healthy and diabetic heart failure subjects stained with hematoxylin and eosin (H&E). The arrowhead shows myocardial cells lacking a nucleus, a key marker of necrotic cells. The cells with stars represent cytoplasmic vacuolization. These cells are surrounded by infiltrating inflammatory cells. **B** Histological analysis of cardiac sections at lower magnifications (8× and 40×). Utilizing H&E (top panel), picrosirius red (middle panel), and Movat pentachrome (bottom panel) stains, these images reveal distinct structural changes in the heart. Diabetic hearts show enhanced fibrotic remodeling with increased collagen (red in picrosirius red), and extensive extracellular matrix disruption with irregular, degenerated myofibers, (visible in Movat pentachrome, with collagen yellow, muscle fibers red, mucin blue, and elastic fibers black); magnifications: 800 µm and 400 µm.

The presence of indicators such as the loss of nuclear details, cytoplasmic vacuolization, and the infiltration of inflammatory cells serves as conclusive evidence of necrotic cells [18]. Necrosis occurs subsequent to cell death, making the identification of necrotic cells a reliable confirmation of cell demise [19]. To ascertain that diabetes indeed instigates myocardial cell death in the failing human heart, we conducted an analysis of H&E-stained histological sections. The observation of cytoplasmic vacuolization (depicted by a “star” symbol) and the absence of the nucleus (indicated by an “arrowhead” symbol) in the diabetic heart failure patients unequivocally demonstrates the presence of myocardial necrotic cells (Fig. 1A). These distinctive features were notably absent in the healthy control heart. Myocardial cell death leads to tissue remodeling and subsequent cardiac fibrosis. Therefore, we assessed features of cardiac remodeling and fibrosis in these samples, finding a marked increase in cardiac remodeling and fibrosis within diabetic hearts (Fig. 1B and S1). Altogether, our findings establish the occurrence of diabetes-induced myocardial cell death in human heart failure.

### Elevated cardiac ferroptosis markers in heart failure patients with diabetes

#### Decreased myocardial GPX4 expression and activity in diabetic heart failure patients

**Fig. 2:**
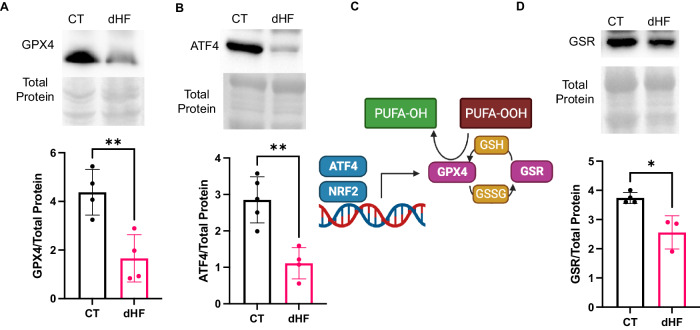
Diabetes downregulates cardiac GPX4 expression and activity in human heart failure. Immunoblotting shows downregulation of cardiac GPX4 (**A**), ATF4 (**B**), and GSR (**D**) in diabetic heart failure. **C** Schematic showing the association of ATF4 and GSR to GPX4 expression and activity and the role of GPX4 in lipid peroxidation. BioRender was used to create this figure. Unpaired two-tailed t-test. *P < 0.05; **P < 0.01. Each point represents one patient. N = 3–5. GPX4 = glutathione peroxidase-4, ATF = activating transcription factor-4, GSR = glutathione reductase, CT = control, healthy subjects, dHF = diabetic heart failure patients.

According to guidelines for evaluating myocardial cell death, diminished expression of glutathione peroxidase-4 (GPX4), a lipid-specific antioxidant, serves as a pivotal indicator of ferroptosis [15]. In line with these guidelines, we assessed myocardial GPX4 levels in both healthy control (CT) subjects and diabetic heart failure (dHF) patients. The observed reduction in cardiac GPX4 in the dHF suggests an elevation in ferroptosis (Fig. 2A). Activating transcription factor-4 (ATF4) serves as an inducer of GPX4 expression and activity [20, 21]. Consequently, we examined ATF4 expression in myocardial tissue of CT and dHF. The decreased levels of myocardial ATF4 in dHF (Fig. 1B), imply that the diminished ATF4 likely contributes to the reduction in GPX4 expression and activity. ATF4 plays a role in upregulating system xc- to biosynthesize glutathione (GSH), crucial for GPX4 activity in reducing lipid hydroperoxides into alcohol [10, 21]. However, GSH undergoes reduction to glutathione disulfide (GSSG) during the process of reducing lipid hydroperoxides. To sustain GPX4 activity, GSSG must be enzymatically reduced back to GSH, a function performed by glutathione reductase (GSR). Consequently, GSR is indispensable for GPX4 activity (Fig. 2C). We further assessed myocardial GSR levels through immunoblotting in both the CT and dHF, revealing decreased GSR in the dHF, indicating decline in GPX4 activity (Fig. 2D). In summary, these findings collectively indicate that the downregulation of ATF4 and GSR in the dHF contributes to the mechanisms leading to reduced expression and activity of GPX4.

#### Impaired iron homeostasis promoting redox-active iron in diabetic heart failure patients

**Fig. 3:**
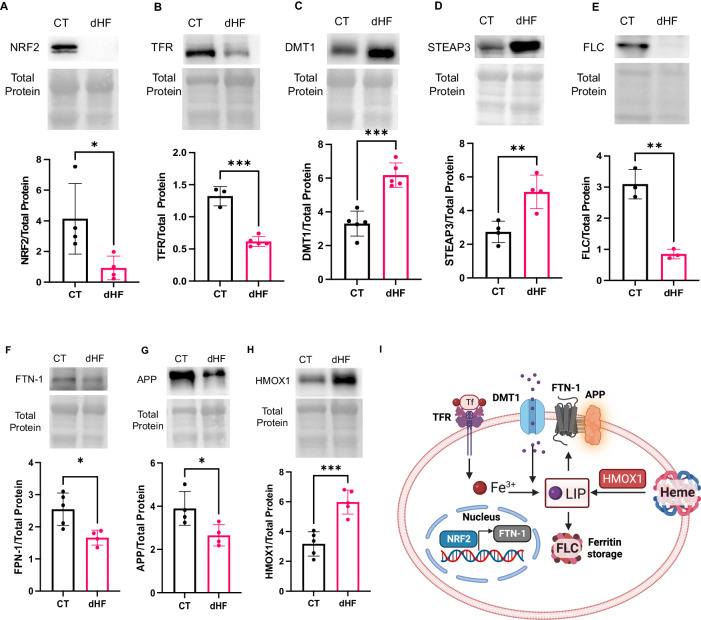
Diabetes impairs iron homeostasis to upregulate cardiac redox-active iron in human heart failure. Immunoblotting shows downregulation of cardiac NRF2 (**A**), TFR (**B**), FLC (**E**), FPN1 (**F**), and APP (**G**) and upregulation of DMT1 (**C**), STEAP3 (**D**), and HMOX1 (**H**) in humans with diabetic heart failure. **I** Schematic showing the association of these molecules with redox-active labile iron pool (LIP) and iron homeostasis. BioRender was used to create this figure. Unpaired two-tailed t-test. *P < 0.05; **P < 0.01; ***P < 0.001. Each point represents one patient. N = 3–5. NRF2 = nuclear erythroid-2-related factor-2, TFR = transferrin receptor, FLC = ferritin light chain, FPN1 = ferroportin-1, APP = beta-amyloid precursor protein, STEAP3 = six-transmembrane epithelial antigen of prostate-3, CT = control, healthy subjects, dHF = diabetic heart failure patients.

Limited studies have investigated iron homeostasis in the diabetic heart [10]. Impaired iron homeostasis can result from increased cellular import and diminished cellular storage and export of iron, potentially leading to an upregulation of redox-active labile iron pool (LIP) and subsequent ferroptosis [10]. In the diabetic heart, nuclear factor erythroid-2-related factor-2 (NRF2) plays a crucial role in regulating iron homeostasis by promoting iron storage in type 1 diabetes mellitus (T1DM) hearts and iron export in type 2 diabetes mellitus (T2DM) hearts [22, 23]. Additionally, NRF2 upregulates GPX4 transcription, further emphasizing its pivotal role in the regulation of cardiac ferroptosis [24]. To assess the impact of NRF2 on cardiac ferroptosis, we conducted immunoblotting to measure NRF2 levels in CT and dHF heart tissues, revealing a downregulation of NRF2 in the hearts of dHF (Fig. 3A). The diminished cardiac NRF2 levels suggest an increase in cardiac ferroptosis in humans with diabetic heart failure.

To unravel the mechanism behind impaired iron homeostasis in dHF, we investigated iron import by assessing the levels of cardiac transferrin receptor (TFR). TFR, situated on the cell membrane, facilitates the import of ferric iron [10]. Immunoblotting results revealed a decrease in cardiac TFR levels in dHF (Fig. 3B). This finding implies that cardiac iron overload in dHF may not be explicitly attributed to an increase in iron import through TFR.

Ferric iron, however, can be imported independently of TFR by the divalent metal transporter-1 (DMT1) [25]. The process involves importing ferric iron into the endosome and converting it into ferrous iron through the six-transmembrane epithelial antigen of prostate-3 (STEAP3). Consequently, we measured cardiac levels of STEAP3 and DMT1 in the CT and dHF hearts. The observed upregulation of DMT1 and STEAP3 in dHF (Fig. 3C, D) suggests that increased iron import through DMT1 and heightened generation of ferrous iron are potential mechanisms contributing to the upregulation of cardiac LIP in dHF.

Elevated LIP levels in dHF may stem from diminished storage of ferrous iron within ferritin and/or reduced iron export through ferroportin-1 (FPN1). Ferritin, comprising both ferritin heavy chain and crucial ferritin light chain (FLC), was examined in cardiac tissue from CT and dHF samples [26]. Immunoblotting results revealed a downregulation of FLC in the dHF (Fig. 3E), indicating that impaired iron storage contributes to the upregulation of LIP in dHF. Similarly, FPN1 was found to be downregulated (Fig. 3F), signifying impaired iron export in the dHF. FPN1 stability at the cell surface levels is influenced by beta-amyloid precursor protein (APP) [27]. In dHF, the cardiac levels of APP were downregulated (Fig. 3G). This finding suggests that the compromised stabilization and reduced expression of FPN1 contribute to impaired iron export in dHF, leading to increased cardiac LIP levels.

Additionally, increased heme metabolism contributes to LIP. The enzyme heme oxygenase-1 (HMOX1), responsible for heme metabolism and ferroptosis, was measured in cardiac tissue from CT and dHF [28]. Immunoblotting revealed an upregulation of HMOX1 in the dHF (Fig. 3H), suggesting that increased heme metabolism contributes to elevated LIP in dHF.

Altogether, these findings unveil that impaired iron homeostasis in dHF is plausibly a results of increased heme metabolism, upregulated conversion of ferric to ferrous iron, diminished storage of ferrous iron into ferritin, and impaired efflux of iron through FPN1. The reduced levels of APP further decrease the stability of FPN1 (Fig. 3I).

#### Increased membrane incorporation of activated PUFA in the hearts of diabetic heart failure patients

**Fig. 4:**
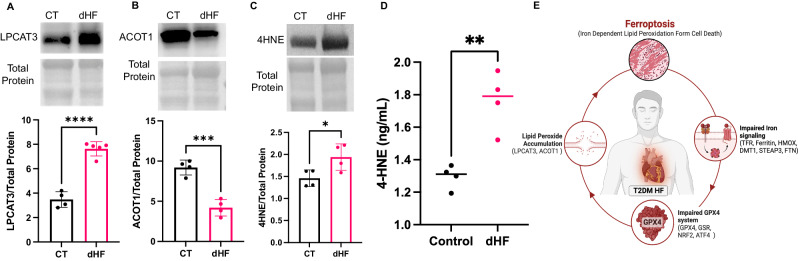
Diabetes promotes cardiac lipid peroxidation in human heart failure. Immunoblotting of heart tissue shows upregulation of LPCAT3 (**A**) and 4HNE (**C**) and its protein adducts (**D**) and downregulation of ACOT1 (**B**) in diabetic heart failure patients. LPCAT3 promotes while ACOT1 prevents lipid peroxidation. 4HNE and its protein adducts demonstrate levels of lipid peroxide with increased 4HNE suggesting ferroptosis. Unpaired two-tailed t-test. *P < 0.05; **P < 0.01; ***P < 0.001; ****P < 0.0001. Each point represents one patient. N = 4–5. LPCAT3 = lysophosphatidylcholine acyltransferase-3, ACOT1 = acyl-CoA thioesterase-1, 4HNE = 4-hydroxynonenal, CT = control, healthy subjects, dHF = diabetic heart failure patients. **E** Schematics showing the mechanisms of cardiac ferroptosis regulation in humans with diabetic heart failure.

A crucial step in the peroxidation of membrane lipids involves the integration of active polyunsaturated fatty acid (PUFA) into membrane phospholipid (PL), forming PUFA–PL. Lysophosphatidylcholine acyltransferase-3 (LPCAT3) plays a vital role in PUFA–PL formation, while acyl-CoA thioesterase-1 (ACOT1) inhibits PUFA–PL formation, preventing ferroptosis [10]. Through immunoblotting, we measured cardiac levels of LPCAT3 and ACOT1 in both CT and dHF heart tissues. The results revealed an upregulation of LPCAT3 and downregulation of ACOT1 in dHF (Fig. 4A, B), suggesting increased PUFA–PL formation and thus elevated lipid peroxidation signaling in the dHF heart. Lipid peroxide levels serve as an indicator of ferroptosis. Therefore, we quantified cardiac lipid peroxide levels in diabetic heart failure patients by measuring 4-hydroxynonenal (4HNE) and its protein adducts (Fig. 4C, D). Elevated levels of both 4HNE and its adducts in the diabetic heart compared to controls indicate increased lipid peroxidation (Fig. 4C, D), further substantiating the occurrence of ferroptosis in the diabetic human heart failure.

Altogether, the observed upregulation of lipid peroxidation signaling and increased levels of lipid peroxides, coupled with elevated labile iron pool (LIP) due to impaired iron homeostasis, and the reduction in antioxidants resulting from diminished expression and activity of glutathione peroxidase-4 (GPX4), collectively establish the occurrence of myocardial ferroptosis in humans with diabetic heart failure (Fig. 4E).

### Novel molecular mechanisms of cardiac ferroptosis in diabetic heart failure

**Fig. 5:**
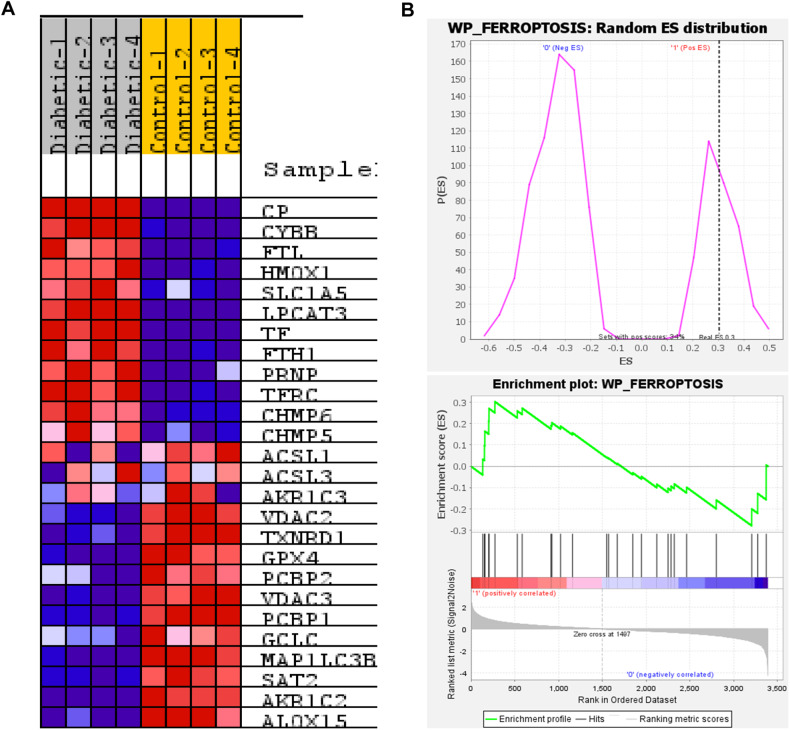
Proteomic and bioinformatic analysis of cardiac tissue in diabetic heart failure patient. **A** Protein expression heatmap illustrating the differential regulation of ferroptosis-associated proteins between diabetic patients and healthy controls (red = increased expression and blue = decreased expression). CP = ceruloplasmin, CYBB = cytochrome b-245 heavy chain, FTL = ferritin light chain, HMOX1 = heme oxygenase-1, SLC1A5 = solute carrier family-1 member 5, LPCAT3 = lysophosphatidylcholine acyltransferase- 3, TF = transferrin, FTH1 = ferritin heavy chain-1, PRNP = major prion protein, TFRC = transferrin receptor protein, CHMP6 = charged multivesicular body protein-6, CHMP5 = charged multivesicular body protein-5, ACSL1 = acyl-CoA synthetase long-chain family member-1, ACSL3 = acyl-CoA synthetase long-chain family member-3, VDAC2 = voltage-dependent anion-selective channel protein-2, TXNRD1 = thioredoxin reductase-1, GPX4 = glutathione peroxidase-4, PCBP2 = poly(C)-binding protein-2, VDAC3 = voltage-dependent anion-selective channel protein-3, PCBP1 = poly(C)-binding protein-1, GCLC = glutamate-cysteine ligase catalytic subunit, MAP1LC3B = microtubule associated protein-1 light chain-3 beta, SAT2 = spermidine/spermine N1-acetyltransferase family member-2, AKR1C2 = aldo-keto reductase family-1 member C2, ALOX 15 = arachidonate 15-lipoxygenase. **B** Visualization of protein distribution and enrichment in ferroptosis pathways, validated through gene set enrichment analysis (GSEA). Sample size: N = 4 for each group.

To uncover novel mechanisms of cardiac ferroptosis, we embarked on a comprehensive examination of molecular pathways in diabetic heart failure patients. We honed in on the critical roles of essential proteins within the ferroptosis signaling cascade. Utilizing advanced proteomic technique liquid chromatography–tandem mass spectrometry (LC–MS/MS) and gene set enrichment analysis (GSEA), we identified several proteins previously unrecognized, which are upregulated in diabetes-induced cardiac ferroptosis. Notably, we pinpointed ceruloplasmin (CP), vital for iron metabolism, and cytochrome b-245 heavy chain (CYBB), an integral component of the NADPH oxidase complex that facilitates the production of reactive oxygen species. Additionally, we documented the downregulation of voltage-dependent anion-selective channel protein-2 (VDAC2), which is crucial for regulating mitochondrial membrane potential to thwart ferroptosis (Fig. 5A). Our findings also included the upregulation of key iron storage proteins such as ferritin light chain (FTL) and ferritin heavy chain-1 (FTH1), coupled with a significant downregulation of glutathione peroxidase-4 (GPX4), a pivotal antioxidant enzyme (Fig. 5A). The proteomic data, alongside the enrichment plots, provided a detailed landscape of the ferroptosis signaling network, underscoring the potential for targeted therapeutic interventions in diabetic heart failure (Fig. 5B).

We also observed inconsistent expression patterns in proteins such as acyl-CoA synthetase long-chain family member 1 (ACSL1) and ACSL3, which are involved in lipid metabolism that can influence ferroptosis through lipid peroxidation (Fig. 5A). This suggests the necessity for a larger dataset to accurately determine their roles in ferroptosis within the diabetic heart.

In summary, the findings collectively demonstrate an upregulation of ferroptosis signaling at multiple levels: through increased PUFA–PL formation, elevated LIP due to impaired iron homeostasis, downregulated GPX4, and heightened myocardial cell death in dHF. This marks the first report establishing cardiac ferroptosis in the human heart and elucidating potential mechanisms underlying diabetes-induced impaired iron homeostasis, downregulated GPX4, and lipid peroxidation in human heart failure. These discoveries highlight the increased susceptibility to ferroptosis in diabetic heart failure.

### Exploring myocardial cell death mechanisms in diabetic heart failure patients

**Fig. 6:**
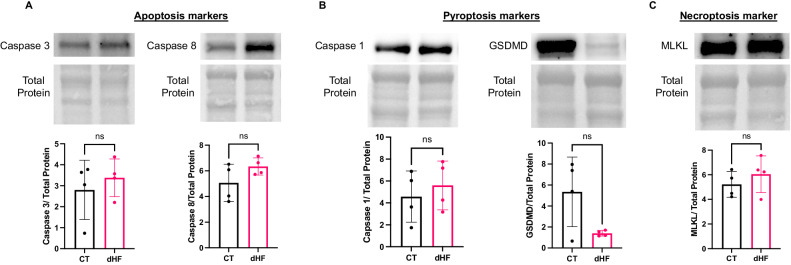
Stability of key non-ferroptotic myocardial cell death markers in diabetic heart failure patient. **A** No change in the expression levels of cardiac caspase 3 and caspase 8, the essential markers of apoptosis in diabetic heart failure patients. **B** Similar cardiac concentrations of caspase 1 and gasdermin D (GSDMD), the key molecular markers of pyroptosis, in the diabetic heart failure patients. **C** No difference in the cardiac levels of mixed lineage kinase domain-like (MLKL), an important marker for necroptosis. Analysis was performed using an unpaired two-tailed t-test. Data points represent individual patients, with a total sample size of N = 4.

In addition to ferroptosis, at least five distinct forms of cell death mechanisms have been identified in cardiac pathology [15]. To assess the involvement of other cell death pathways in myocardial cell death among diabetic heart failure patients, we analyzed several molecular markers associated with apoptosis, pyroptosis, and necroptosis in both diabetic heart failure patients and control subjects (Fig. 6). Our observations revealed that the levels of caspase 3 and caspase 8, which are key markers of apoptosis, remained unchanged in the diabetic heart (Fig. 6A). Similarly, caspase 1 and gasdermin D, an essential markers of pyroptosis, also showed no significant variation (Fig. 6B). Furthermore, there were no detectable changes in the cardiac levels of mixed lineage kinase domain-like protein (MLKL), an important marker of necroptosis, in the diabetic heart (Fig. 6C). The stability of these cell death mechanisms suggests that ferroptosis may play a dominant role in myocardial cell death in patients with diabetic heart failure.

### Other markers of heart failure increased in dHF

**Fig. 7:**
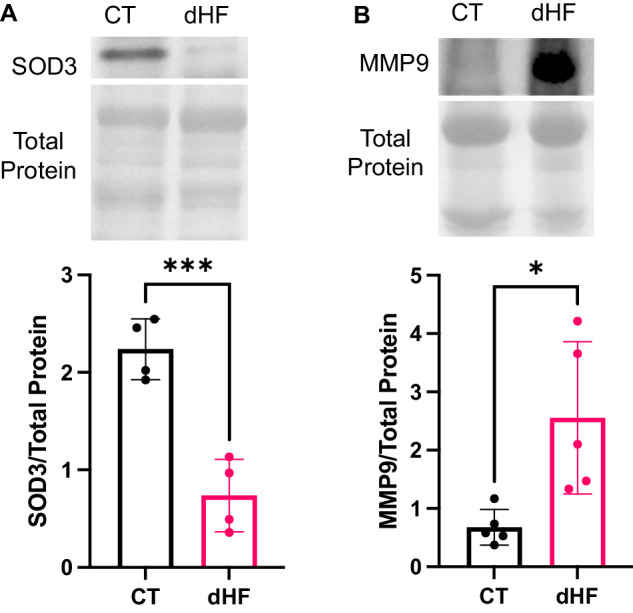
Upregulated molecular markers of heart failure in diabetic heart failure patient. Immunoblotting shows downregulation of cardiac SOD3 (**A**) and upregulation of MMP9 (**B**) in diabetic heart failure. Downregulated SOD3 and upregulated MMP9 are molecular markers of heart failure. Unpaired two-tailed t-test. *P < 0.05; ***P < 0.001. Each point represents one patient. N = 4–5. SOD3 = superoxide dismutase-3, MMP9 = matrix metalloproteinase-9, CT = control, healthy subjects, dHF = diabetic heart failure patients.

To validate our molecular findings, we examined established markers of heart failure in both CT and dHF samples. The diminished expression of the antioxidant superoxide dismutase-3 (SOD3) is linked to an elevated risk of heart failure in diabetic patients [29]. Our results demonstrated a reduction in cardiac SOD3 levels in the dHF (Fig. 7A). The increased circulating levels of matrix metalloproteinase-9 (MMP9) is another marker of heart failure, including diabetes-induced heart failure [30, 31]. Elevated MMP9 levels are associated with both T1DM and T2DM [32]. Therefore, we assessed MMP9 levels through immunoblotting, revealing increased cardiac levels in dHF (Fig. 7B). This further supports the evidence for cardiac pathogenesis at the molecular level for diabetes-induced heart failure.

## Discussion

In the present study, we establish cardiac ferroptosis as a significant contributor to myocardial cell death in human heart failure, particularly among diabetic patients. We identify several novel molecular markers of diabetes-induced ferroptosis and demonstrate its predominant role over other myocardial cell death mechanisms such as apoptosis, pyroptosis, and necroptosis. This research provides the first comprehensive evidence of cardiac ferroptosis in the human heart and elucidates how diabetes intensifies this process. Specifically, we highlight three major pathways implicated in ferroptosis: the activation of polyunsaturated fatty acids (PUFAs) and their integration into membrane lipids, the oxidation of these lipids by the labile iron pool (LIP) to generate lipid peroxides, and the impaired function of glutathione peroxidase-4 (GPX4) in preventing lipid peroxide formation [10, 11]. Together, these pathways play crucial roles in driving cardiac ferroptosis in humans with diabetic human heart failure. These insights offer a deeper understanding of how ferroptosis contributes to cardiac dysfunction in diabetes.

Following guidelines on myocardial cell death, the pivotal markers of cardiac ferroptosis include the downregulation of GPX4 and the upregulation of LIP [15]. Our results not only demonstrate the concomitant downregulation of GPX4 and upregulation of molecular pathways increasing LIP in the heart of diabetic patients but also unveil the potential mechanisms behind the diminished expression and activity of GPX4 in diabetic human hearts. Diabetes, in this context, downregulates ATF4 to reduce GPX4 expression and suppresses GSR to decrease GPX4 activity in the human heart. Furthermore, diabetes disrupts iron homeostasis by enhancing iron import through DMT1, inhibiting iron storage into ferritin (FLC), and impeding iron export through FPN1. Our investigation revealed that TFR may not be explicitly implicated in cardiac iron import in diabetic heart failure patients and rather iron import is facilitated by DMT1. Additionally, the decreased levels of APP emerged as a plausible mechanism for reduced iron efflux through FPN1 in diabetic human hearts. Moreover, the upregulation of LPCAT3 and downregulation of ACOT1 contribute to the increased incorporation of activated PUFA into membranes in diabetic human hearts. The increased cardiac levels of 4HNE, a lipid peroxide, further support cardiac ferroptosis in humans with diabetic heart failure. This discovery underscores the involvement of lipid peroxidation signaling in the pathogenesis of diabetic heart failure, reinforcing the role of ferroptosis in this context. Our findings on the molecular pathways underlying diabetes-induced ferroptosis in the human heart are valuable for developing therapeutic interventions and may offer novel strategies for managing heart failure in diabetic patients.

Our proteomic analysis of cardiac tissues significantly enhances the understanding of ferroptosis mechanisms in the context of diabetic heart failure. The upregulation of ceruloplasmin (CP) and ferritin (FTL and FTH1) observed in our study aligns with recent findings that underscore the importance of iron accumulation in ferroptosis. This complements the discovery of GPX4’s protective role against lipid peroxidation, a key event in ferroptosis. Our novel observation of the inconsistent expression of acyl-CoA synthetase long-chain family members (ACSL1 and ACSL3) adds a new dimension to the lipid metabolism aspect of ferroptosis. Contrasting with established research, our data suggest that targeting these specific proteins could represent a new therapeutic strategy for managing diabetic heart failure. The novelty of our study lies in the detailed mapping of ferroptosis-related protein changes in the diabetic heart, which may drive the development of innovative treatments for diabetes-induced myocardial ferroptosis.

While diabetes has been linked to an elevated risk of heart failure, there has been a dearth of evidence supporting diabetes-induced cell death in the human heart [1]. Our histological analysis, however, uncovered a heightened occurrence of cell death in diabetic heart failure patients, characterized by nuclear loss, cytoplasmic vacuolization, and presence of infiltrating inflammatory cell. The absence of these features in healthy hearts substantiates the association between diabetes and increased myocardial cell death, definitively confirming the induction of myocardial necrosis in diabetic heart failure patients. Increased myocardial cell death by diabetes is bolstered by the presence of cardiac remodeling and fibrosis in the diabetic heart failure patients.

Our results find further support in the decreased levels of antioxidant SOD3 and increased expression of MMP9, both established markers of heart failure. This corroboration serves to reinforce the molecular evidence for diabetes-induced heart failure and its associated pathological mechanisms.

Recognizing the molecular variability evident in diabetic heart failure patients [17], we methodically structured our experiment, meticulously matching age, sex, and race between the diabetic and healthy cohorts. This rigorous approach not only allowed us to diminish potential confounding factors contributing to observed variability but also provided a more focused exploration of the specific effects induced by diabetic heart failure.

In conclusion, our findings make a substantial contribution to unraveling the intricacies of cardiac ferroptosis in diabetic human hearts. This paves the way for targeted interventions aimed at alleviating myocardial cell death and enhancing clinical outcomes from individuals with diabetic heart failure.

### Limitations

Like all studies, ours also have few limitations. Due to limited human samples, we have used only male diabetic heart failure patients. Women are at higher risk of diabetes-induced heart failure [33], thus a future thorough study with both men and women diabetic heart failure patients will be needed. In the present study, we have used only two groups, diabetic heart failure and healthy subjects. It is difficult to delineate the effects of diabetes on heart failure with only these two groups. Including a third group of non-diabetic heart failure will elucidate the specific effects of diabetes on myocardial ferroptosis. Although our histological analysis demonstrates myocardial cell death in the diabetic heart failure patients, electron micrography on these tissues are needed to visualize cellular phenotype of ferroptosis, such as reduced mitochondrial volume, increased bilayer membrane density, and reduction or disappearance of mitochondrial cristae [34].

## Materials and methods

### Sample acquisition and patient information

Human heart tissue samples were procured from BioIVT Biospecimens and Research Services. Detailed information regarding the de-identified patients, including age, sex, and other relevant demographics is presented in Table 1. The case IDs are 8845 (specimen IDs: 8365B1, 8365D1, 8364A1, 8365C1, and 8366A1), 52746 (specimen ID: 1137947F), 94499 (specimen ID: 1223740F), and 108082 (specimen IDs:1203605F, 1203621F, and 1203622F). The sample size was estimated based on our previous studies with heart tissues from diabetic heart failure patients [17]. All methods were carried out in accordance with relevant guidelines and regulations.

### Tissue histological analysis

Human tissue samples were fixed in 4% formalin for 2 h, followed by washing and storage in 70% ethanol. Histological and immunohistochemical staining, including hematoxylin and eosin (H&E), sirius red, and Movat pentachrome, were performed at the core facility of the University of Nebraska Medical Center (UNMC). For H&E staining, slides were rehydrated through a graded alcohol series and stained with hematoxylin for nuclear staining. Differentiation was achieved with Focus 0.5% Acid Alcohol, followed by bluing with Vintage Bluing Reagent, and counterstaining with Reserve Eosin Multichrome. Slides were dehydrated through an ascending alcohol series, cleared in xylene, and cover-slipped with permanent mounting medium.

For picrosirius red staining, slides were deparaffinized in xylene, hydrated through a graded alcohol series to distilled water, and stained in picrosirius red solution (0.5 g Direct Red 80 in 500 mL picric acid) for 1 h. After washing in 0.05% acetic acid (five quick dips), the slides were dehydrated, cleared, and cover-slipped for observation under a microscope.

Movat pentachrome staining was conducted following a published protocol [35, 36]. Slides were deparaffinized and rehydrated before staining in Verhoeff’s elastic stain for 15 min, followed by differentiation in 2% ferric chloride until elastic fibers were sharply defined. The sections were then immersed in 5% sodium thiosulfate for 1 min, 3% acetic acid without rinsing, 1% alcian blue solution for 15 min, and crocein scarlet-acid fuchsin solution for 2 min. After immersion in 1% acetic acid (five dips, 1–2 s each), the slides were stained in 5% phosphotungstic acid for 2 min before being dehydrated through absolute alcohol and xylene. Finally, the slides were cover-slipped using permanent mounting media.

### Tissue homogenization

Approximately 25 mg of heart tissue from each human were homogenized in radioimmunoprecipitation assay (RIPA) buffer with protease inhibitor. This process was carried out using a Bead Ruptor 12 homogenizer (Fisher Scientific) for efficient and consistent tissue homogenization.

### Protein estimation

Following homogenization, protein concentration in the human heart tissues was determined using the Pierce BCA Protein Assay kit (Thermo Fisher Scientific #A55865). This assay allows for accurate quantification of total protein.

### SDS–PAGE and immunoblotting

We followed standard immunoblotting (or western blotting) protocol [37]. In brief, a total of 25 μg of protein from each sample was loaded onto SDS–PAGE gels for separation. The proteins were then transferred onto nitrocellulose membranes for blotting. The membranes were blocked in 5% milk for 1 h to prevent non-specific binding. We used total protein (Ponceau staining) for loading control following the guidelines on antibody use in physiological studies [38]. The incubation with primary antibodies was performed at 4 °C overnight, ensuring optimal binding. The secondary antibody incubation was conducted at room temperature for 1 h. The antibodies used for the western blot detection were as follows: GPX4 (ab125066), ATF4 (10835-1-AP), GSR (18257-1-AP), NRF2 (16396-1-AP), TFR (17435-1-AP), STEAP3 (Thermo Fisher PA5-20406), DMT1 (20507-1-AP), FLC (10727-1-AP), FPN1 (26601-1-AP), APP (14-9749-82), HMOX1 (10701-1-AP), LPCAT3 (67882-1-Ig), ACOT1 (ab133948), SOD3 (14316-1-AP), MMP9 (10375-2-AP), anti-4-hydroxynonenal antibody (ab46545), caspase 3 (CST: #9662), caspase 1 (CST: #2225), caspase 8 antibody (ab25901), GSDMD (ProteinTech 66387-1-Ig), and MLKL (ProteinTech 66675-1-Ig). The primary antibodies were used at a dilution of 1:1000, while the secondary antibodies were diluted at 1:2000. The western blots were visualized and imaged using a Bio-Rad ChemiDoc imager. Western blot analysis using BioRad Image Lab software.

### 4HNE ELISA assay

Proteins from human heart tissue were isolated using a homogenizer in RIPA buffer with protease/phosphatase inhibitor. The homogenates were centrifuged for 10 min at 12,000 × *g* at 4 °C. The protein concentration was determined by BCA assay (Pierce™ BCA Protein Assay) and a total of 16 µg homogenates in 50 µL buffer was used for the 4HNE assay. The assay was performed following the manufacture instructions (Abcam # ab238538).

### LC–MS/MS proteomic analysis of human hearts

Tandem mass tag (TMT) proteomics was utilized for profiling proteins in human heart tissues, with mass spectrometry services provided by Creative Proteomics. The protocol included meticulous sample preparation, precise protein quantification, enzymatic digestion with trypsin, labeling with TMT, and strategic sample pooling and fractionation. The samples were then subjected to nano liquid chromatography with tandem mass spectrometry (LC–MS/MS) analysis on a state-of-the-art Q Exactive HF MS/MS platform. Bioinformatic analysis was carried out by the University of Nebraska Medical Center Bioinformatics Core, employing gene set enrichment analysis (GSEA) via version 4.3.2 of the GSEA software, targeting the C2 curated gene sets from the MSigDB [39, 40]. This dual-technique approach enabled an in-depth evaluation of the proteomic alterations associated with cardiac ferroptosis.

### Statistical analysis

The data are presented as mean ± SEM (standard error of mean). Each human is represented as one point to show the variability within the cohort. Unpaired two-tailed t-test was used to evaluate statistical significance between the groups. A P value < 0.05 is considered statistically significant. Statistical analysis was performed using Prism 10 software.

### Supplementary information

Fig. S1 legend

Fig S1

Original Data

## Data Availability

All the raw data are available at the University of Nebraska Medical Center OneDrive and shared drive.
