# Supplementary material for: Regulation of cardiac ferroptosis in diabetic human heart failure: uncovering molecular pathways and key targets
Source: Cell Death Discov. 2024 Jun 1;10:268. doi: 10.1038/s41420-024-02044-w (PMC11144210; doi:10.1038/s41420-024-02044-w)
Supplement: Supplementary file 1 — Fig. S1 legend [file 41420_2024_2044_MOESM1_ESM.docx]

**Supplementary Figure Legend:**

**Fig. S1 Histopathological analysis of cardiac tissues from healthy control subjects and diabetic heart failure patients**. Hematoxylin and Eosin (H & E), Picrosirius Red, and Movat Pentachrome staining reveals distinct structural alterations associated with ferroptosis. Control hearts exhibit normal myofiber architecture, minimal fibrosis, and organized myocardial structure. In contrast, diabetic heart failure patient’s hearts display significant fibrotic remodeling with increased collagen deposition (red, Picrosirius Red), and irregular, degenerated myofibers, extracellular matrix remodeling indicative of disrupted connective tissue (Movat Pentachrome). In Movat Pentachrome staining, collagen is stained yellow, muscle fibers appear red, mucin is blue, and elastic fibers are black. Magnification is: 800 µm, 400 µm, and 60 µm.
