# Supplementary figures and images for: Regulation of cardiac ferroptosis in diabetic human heart failure: uncovering molecular pathways and key targets
Source: Cell Death Discov. 2024 Jun 1;10:268. doi: 10.1038/s41420-024-02044-w (PMC11144210; doi:10.1038/s41420-024-02044-w)

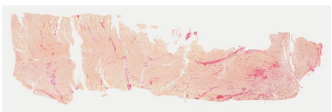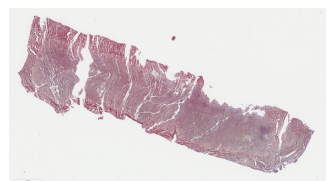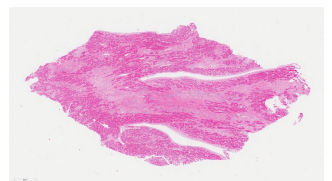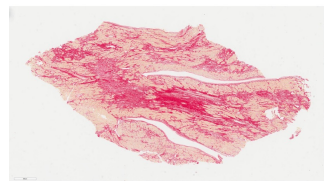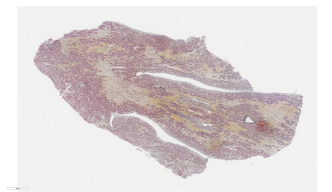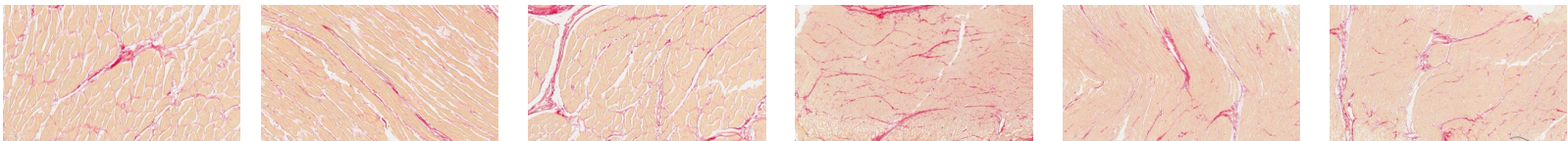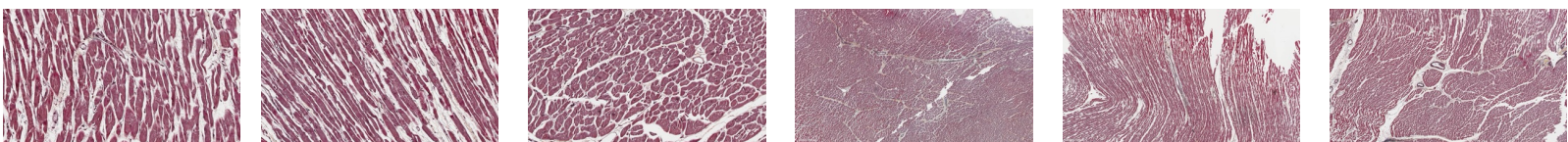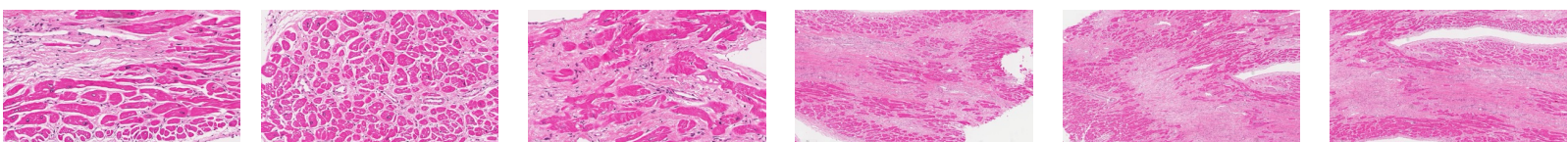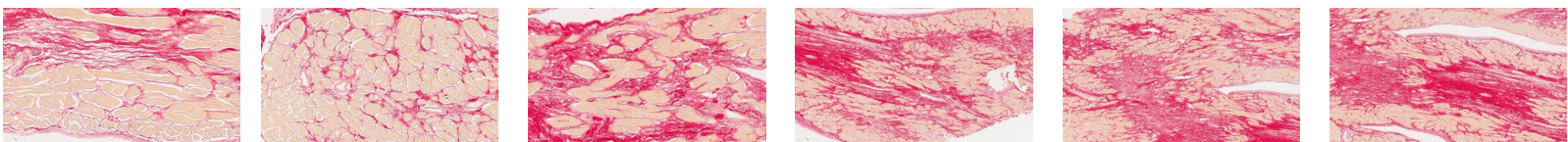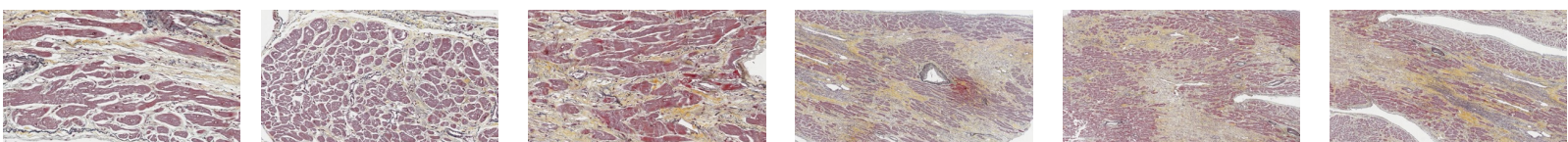

Supplement: Supplementary file 2 — Fig S1 [file 41420_2024_2044_MOESM2_ESM.pdf]
