# Supplementary material for: Regulation of cardiac ferroptosis in diabetic human heart failure: uncovering molecular pathways and key targets
Source: Cell Death Discov. 2024 Jun 1;10:268. doi: 10.1038/s41420-024-02044-w (PMC11144210; doi:10.1038/s41420-024-02044-w)
Supplement: Supplementary file 3 — Original Data [file 41420_2024_2044_MOESM3_ESM.pptx]

## Slide 1
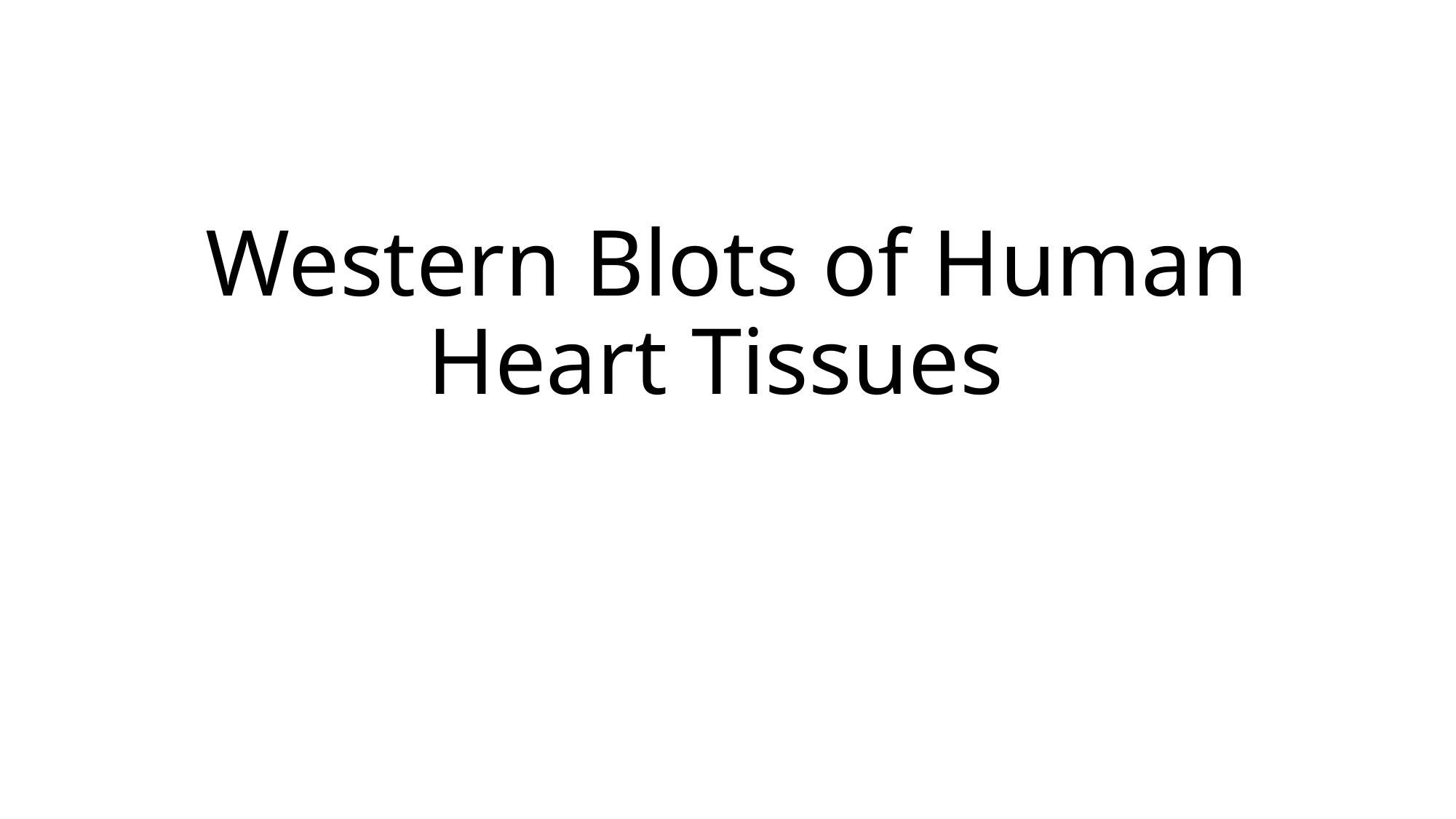

# Western Blots of Human Heart Tissues

## Slide 2
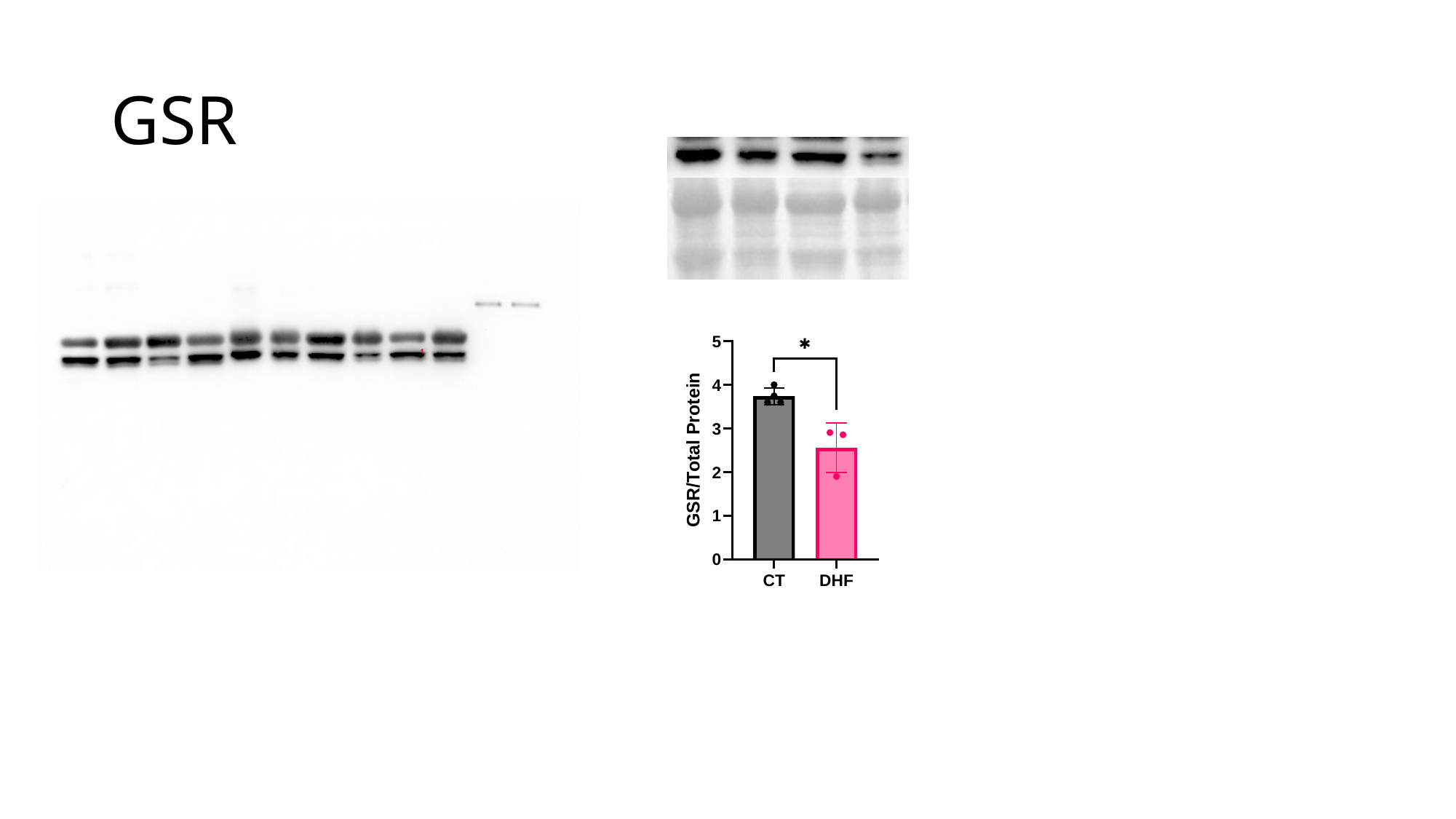

# GSR

## Slide 3
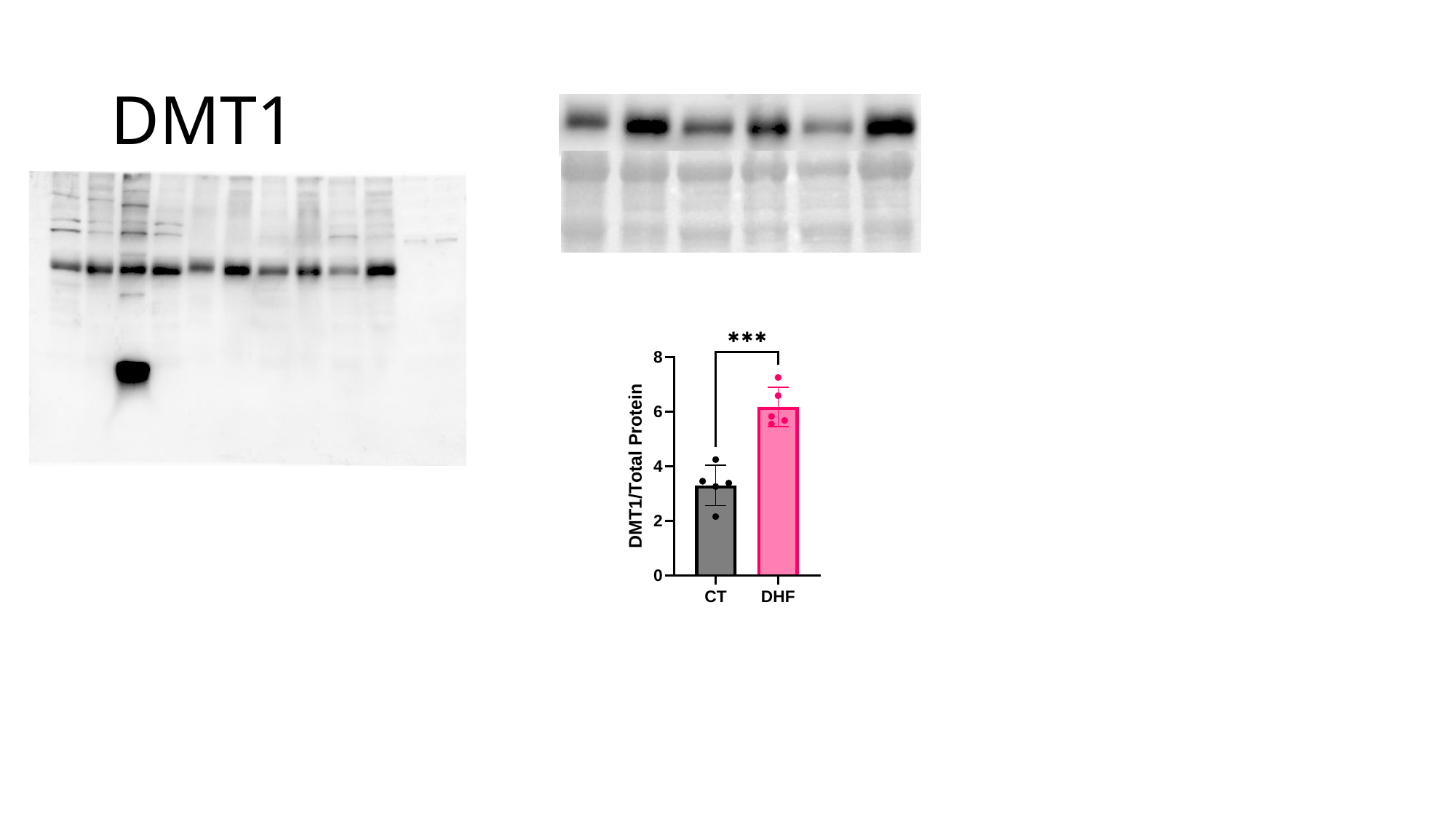

# DMT1

## Slide 4
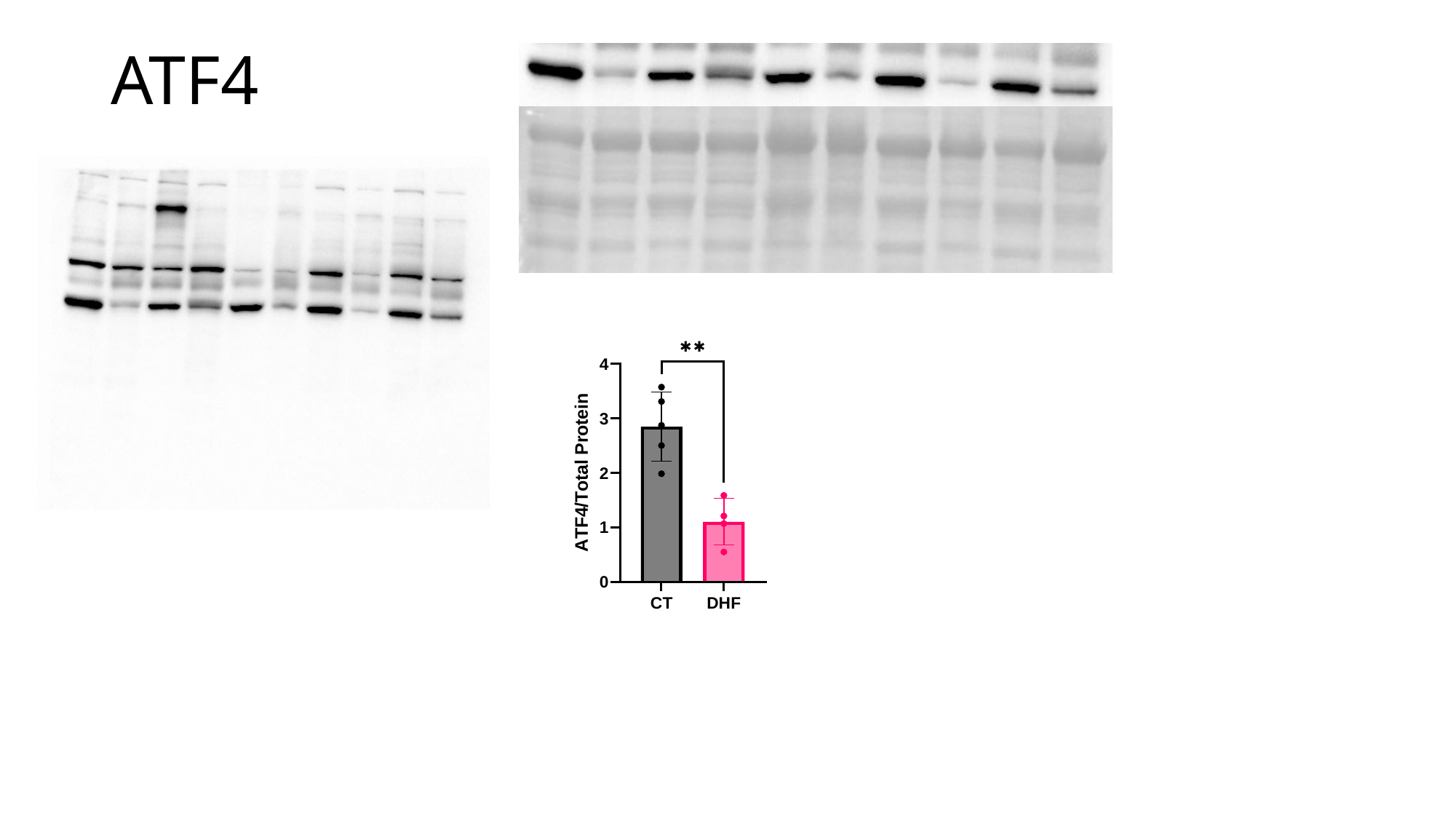

# ATF4

## Slide 5
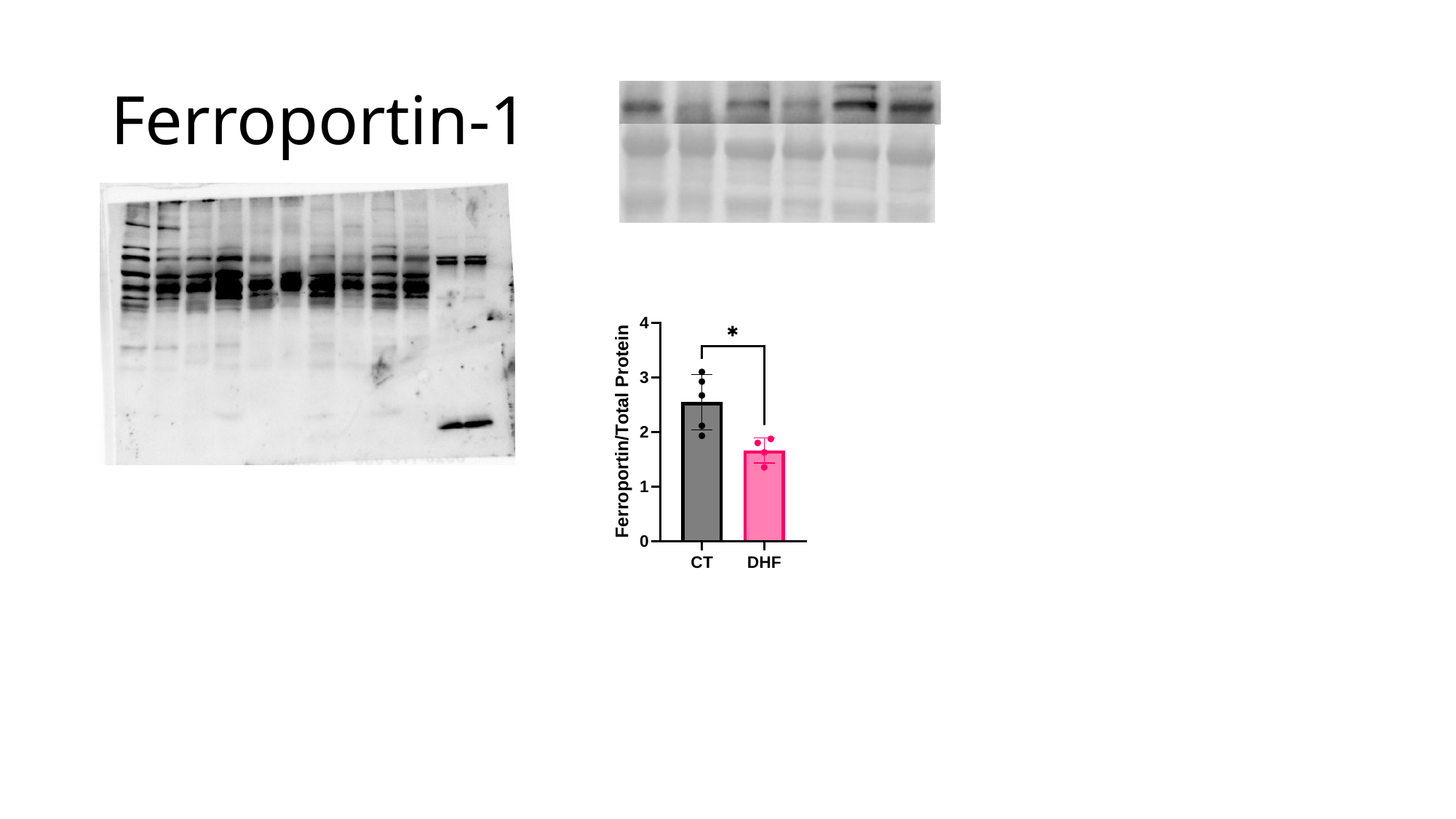

# Ferroportin-1

## Slide 6
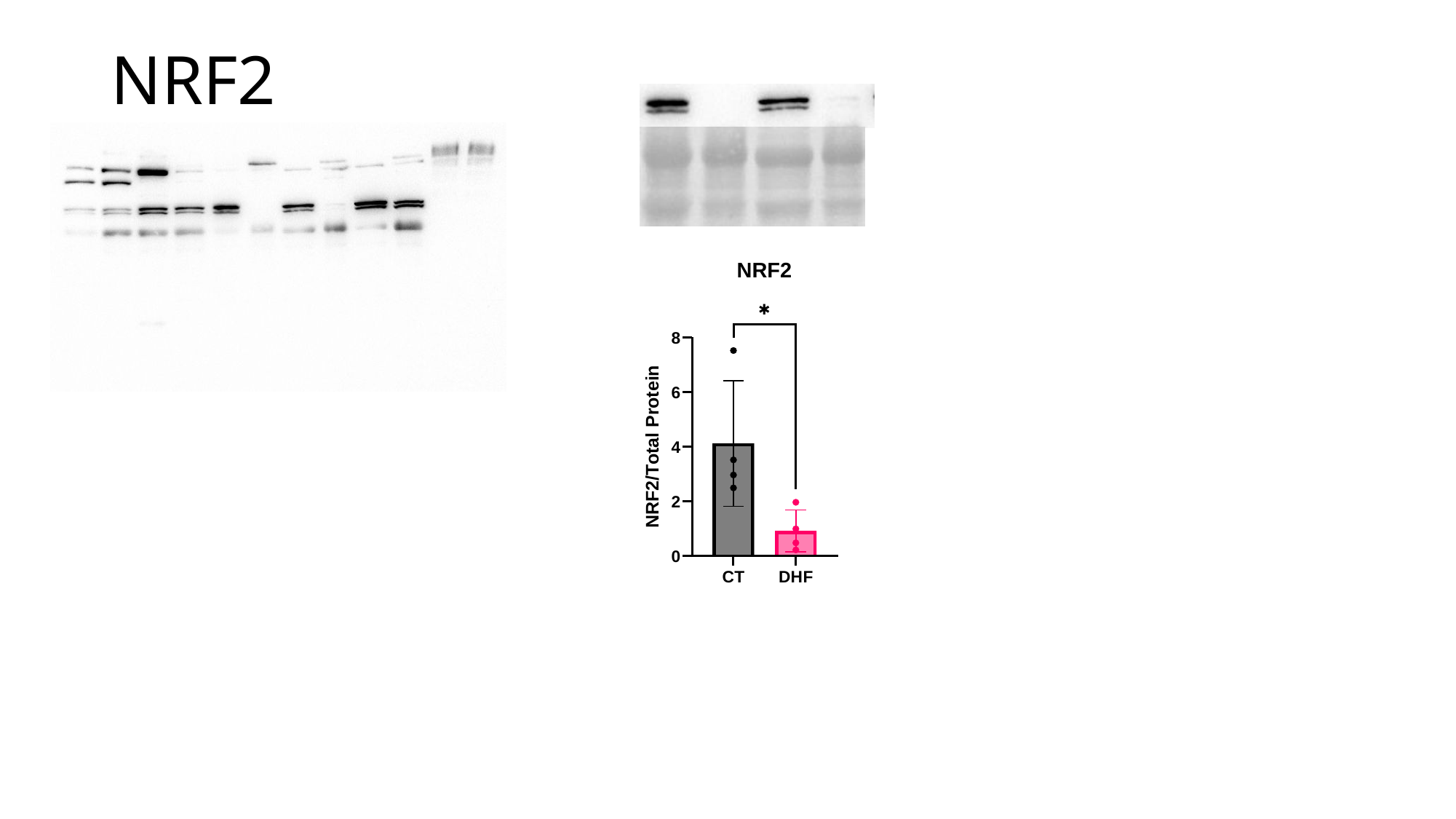

# NRF2

## Slide 7
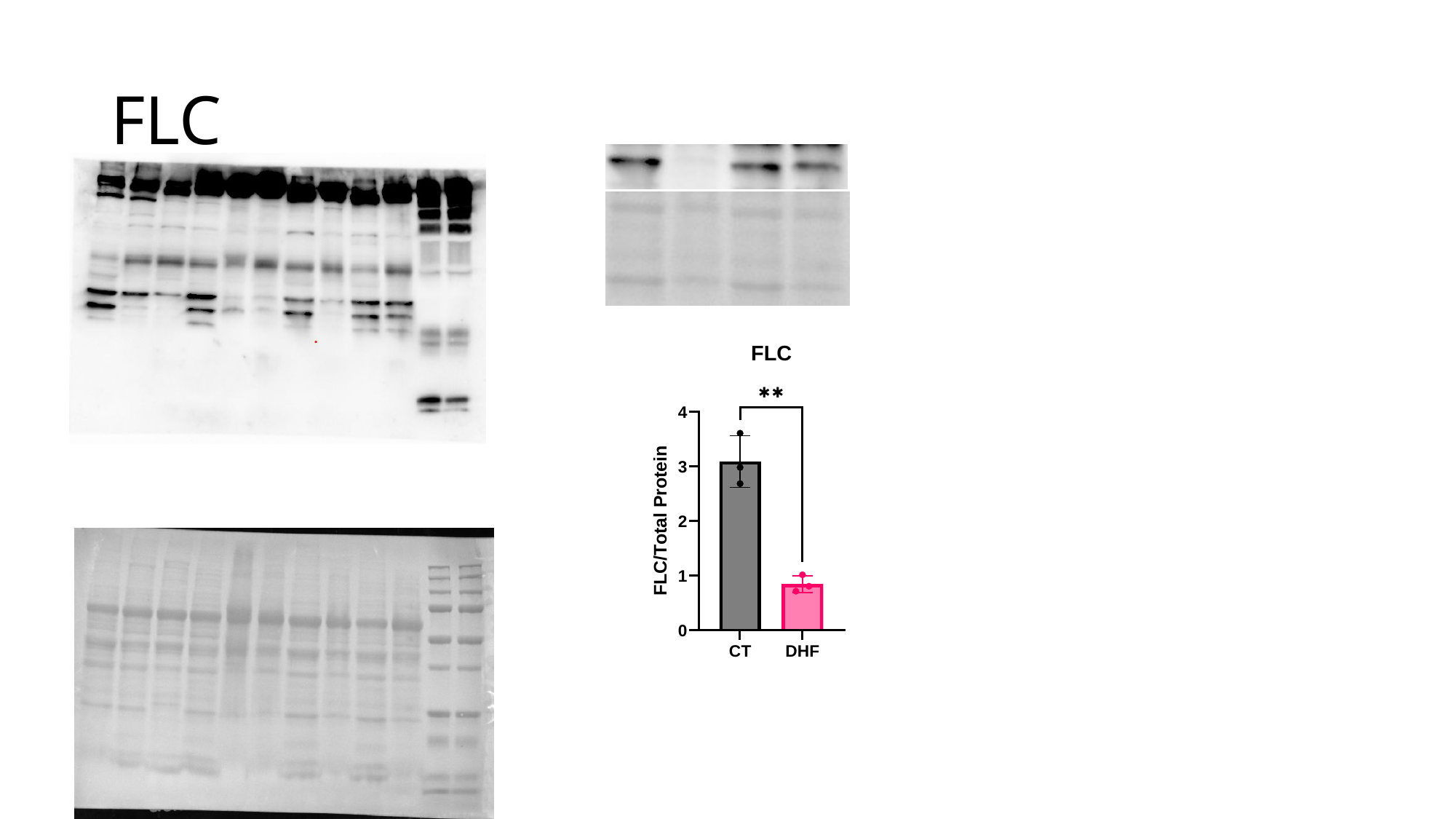

# FLC

## Slide 8
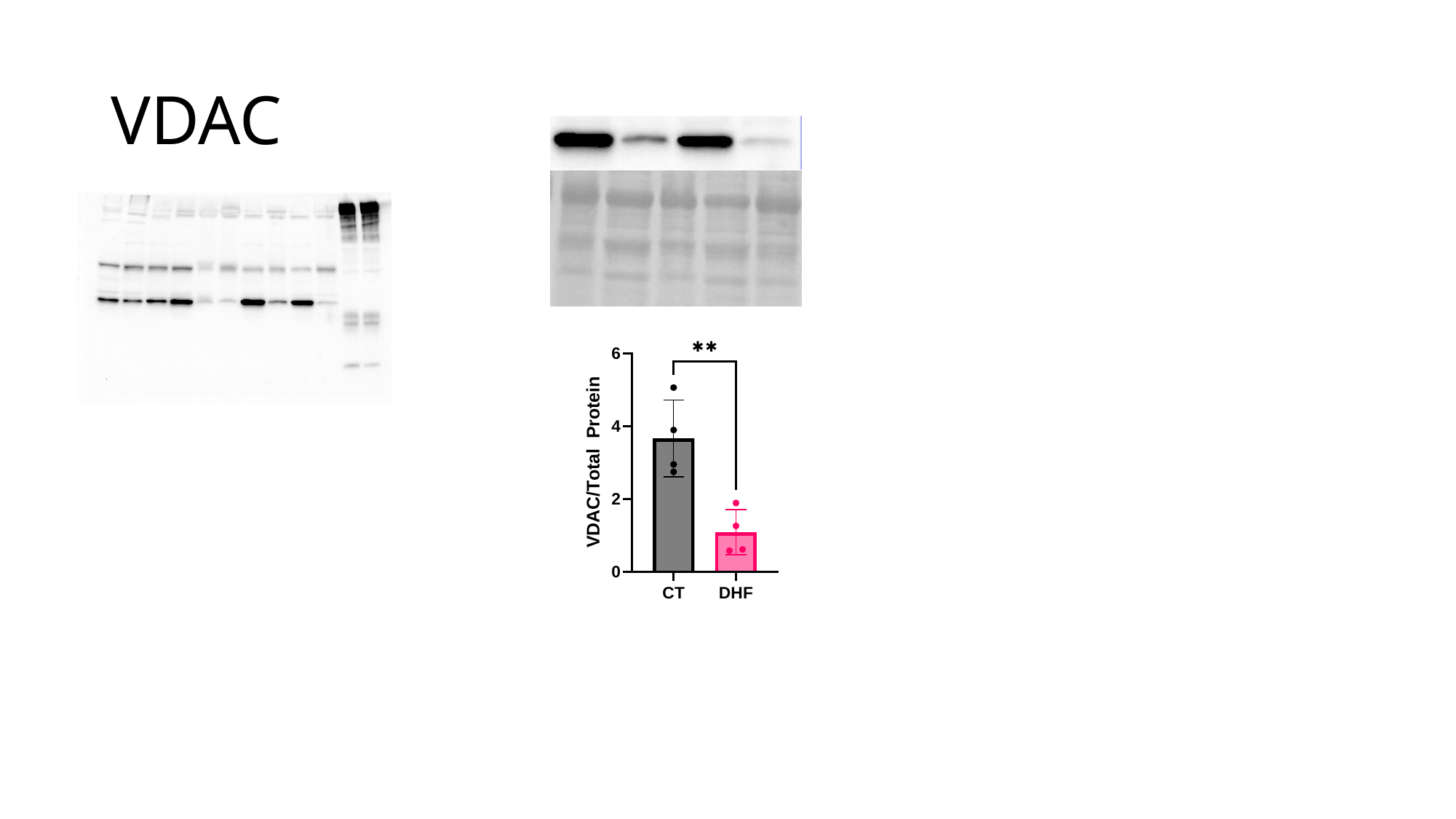

# VDAC

## Slide 9
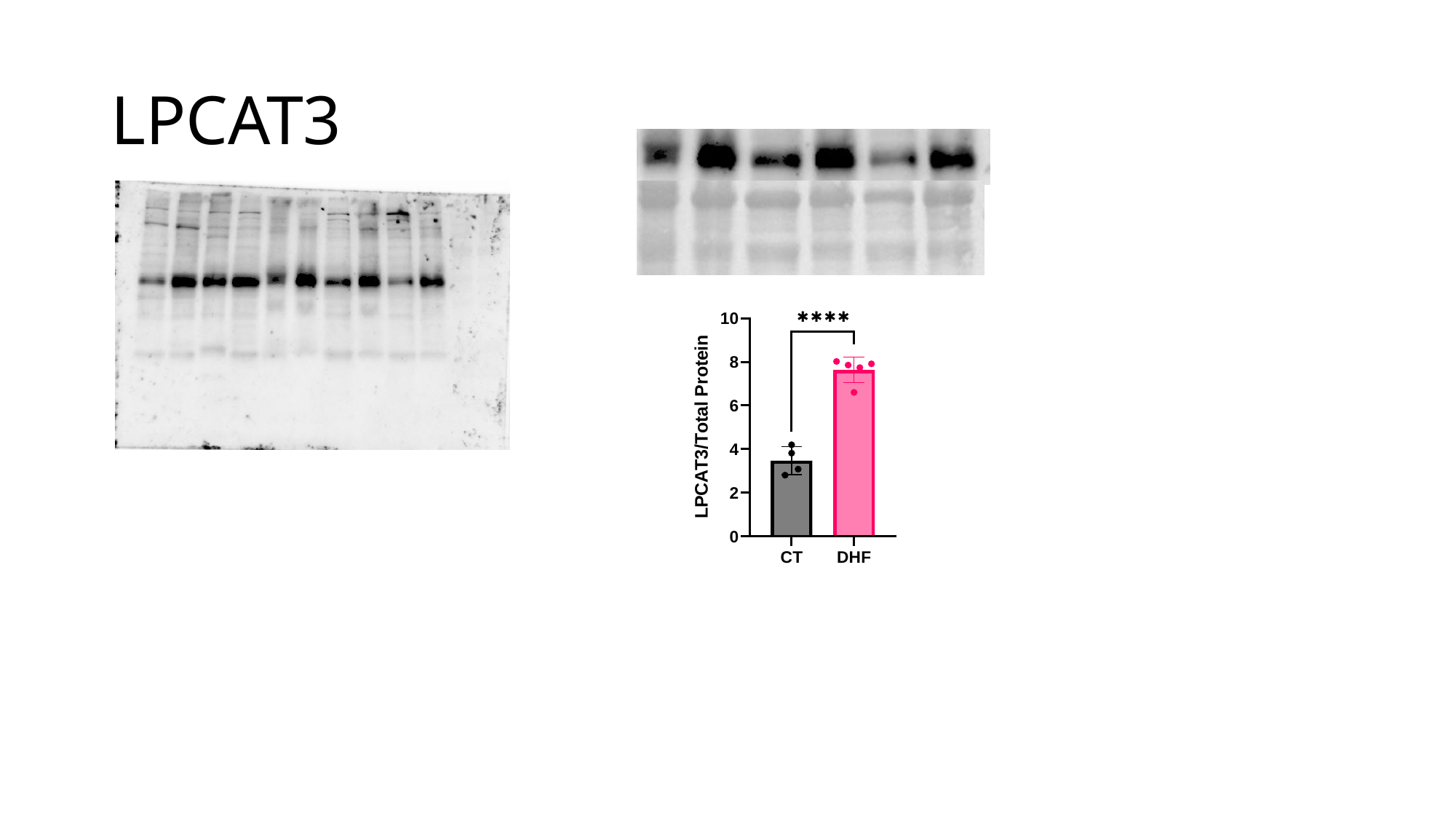

# LPCAT3

## Slide 10
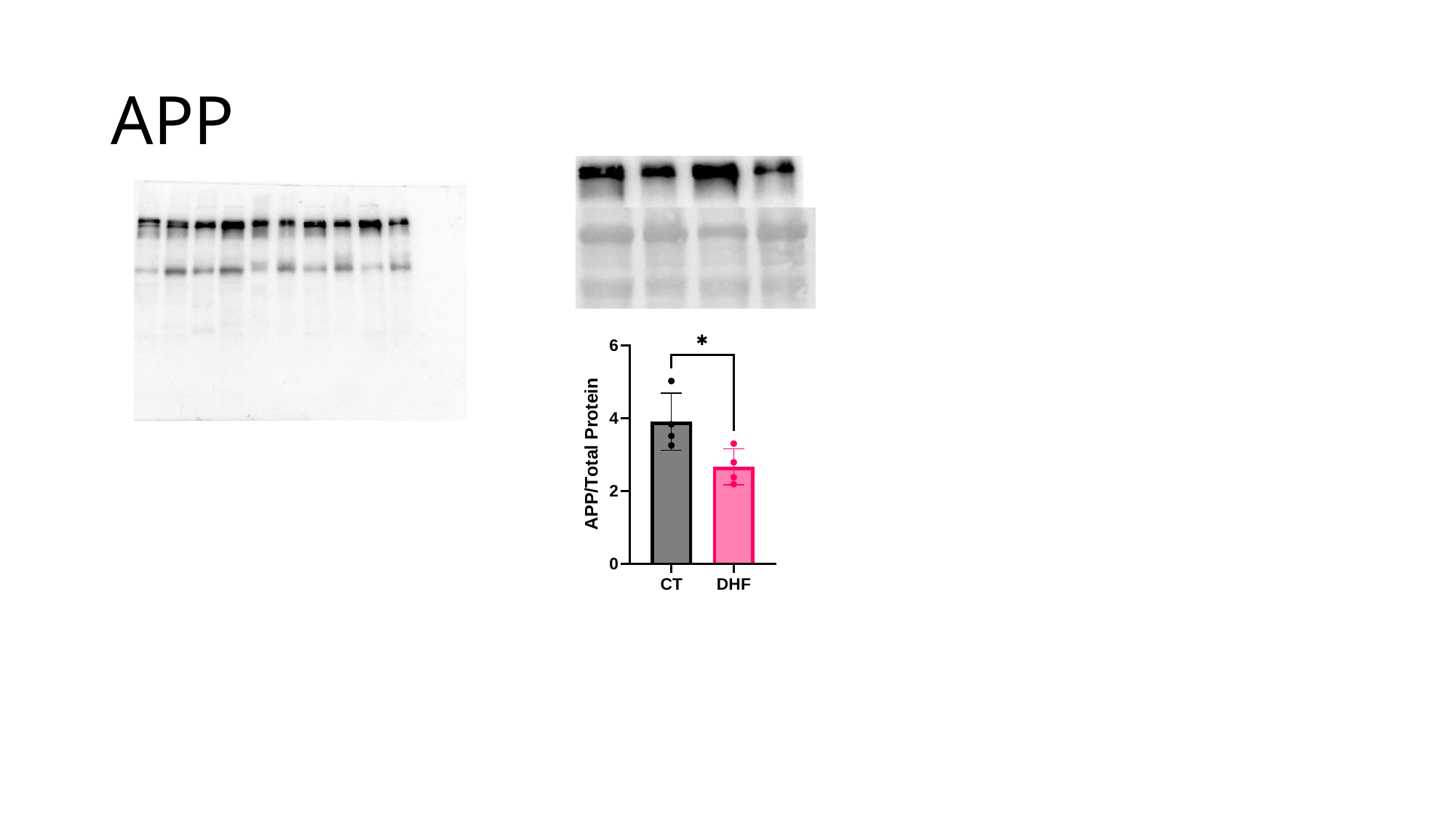

# APP

## Slide 11
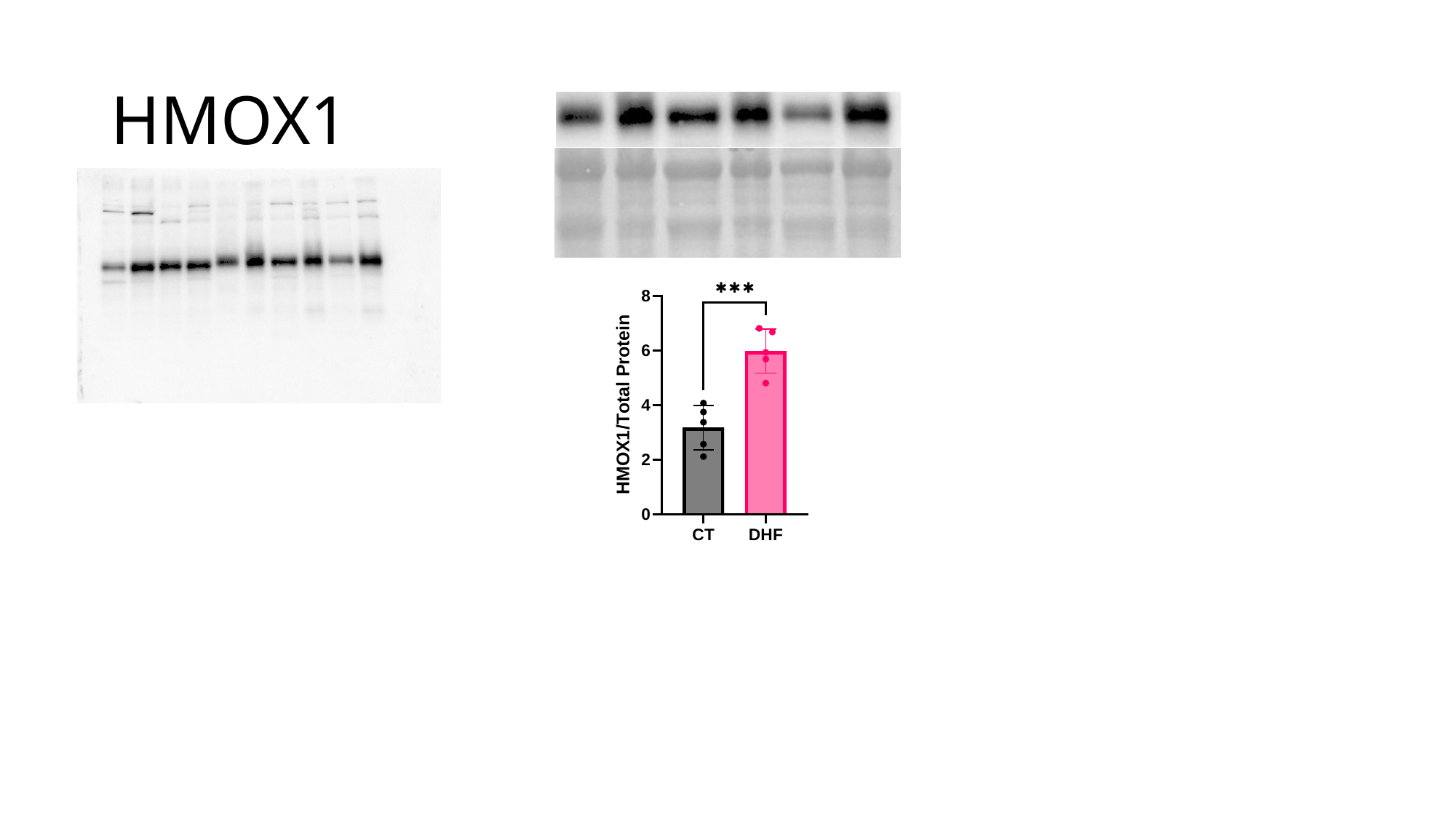

# HMOX1

## Slide 12
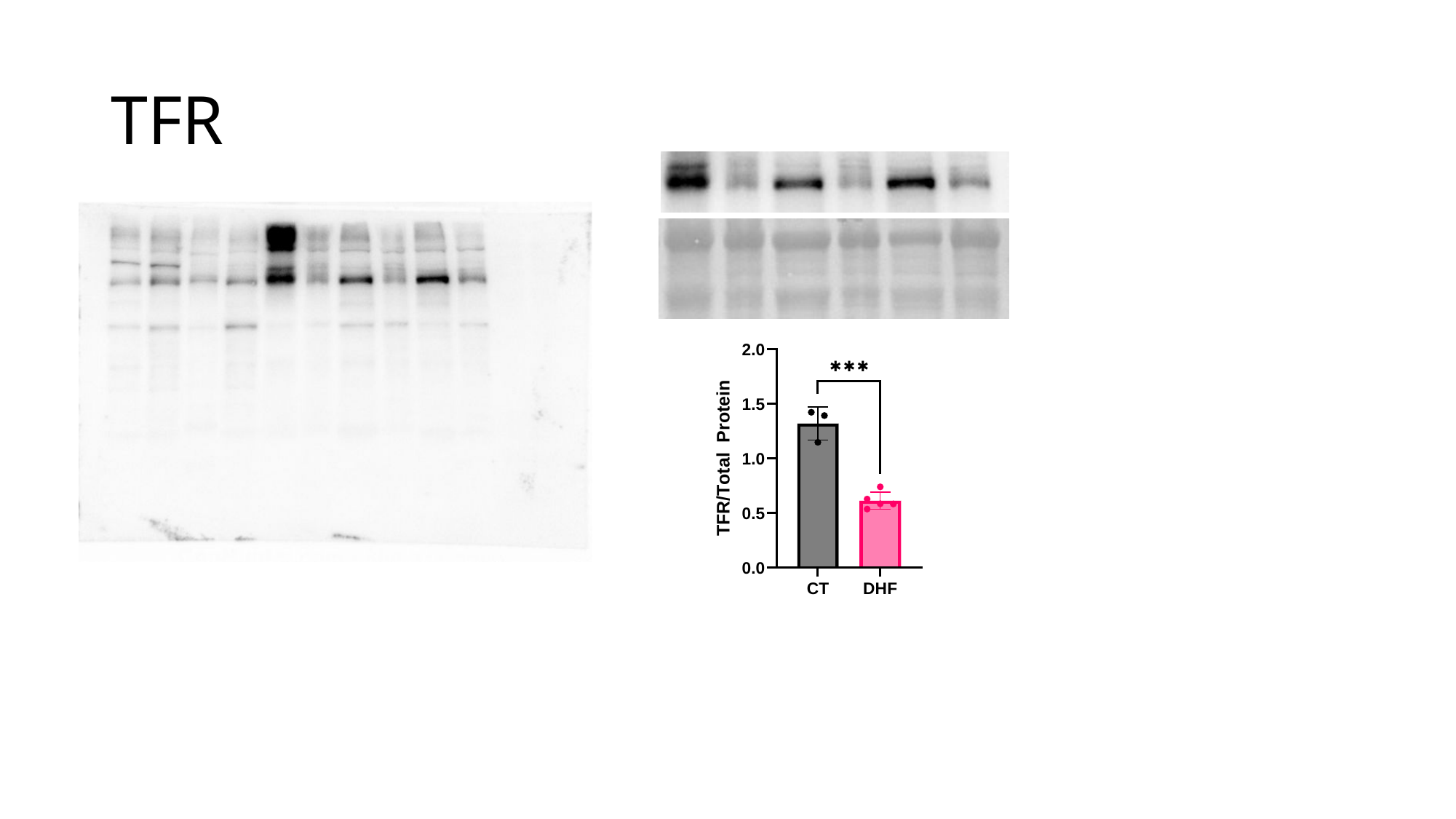

# TFR

## Slide 13
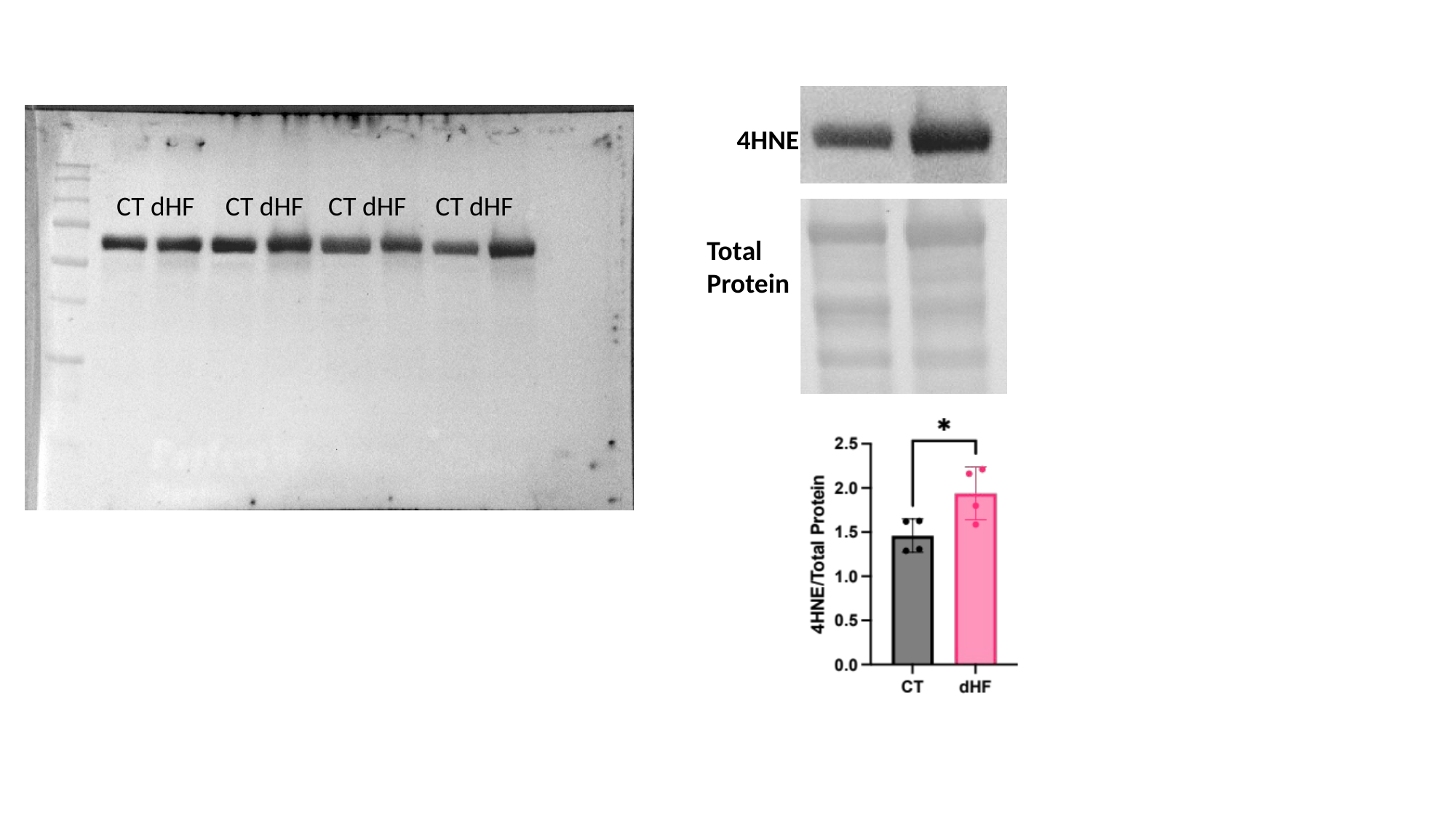

CT dHF
CT dHF
CT dHF
CT dHF
4HNE
Total Protein

## Slide 14
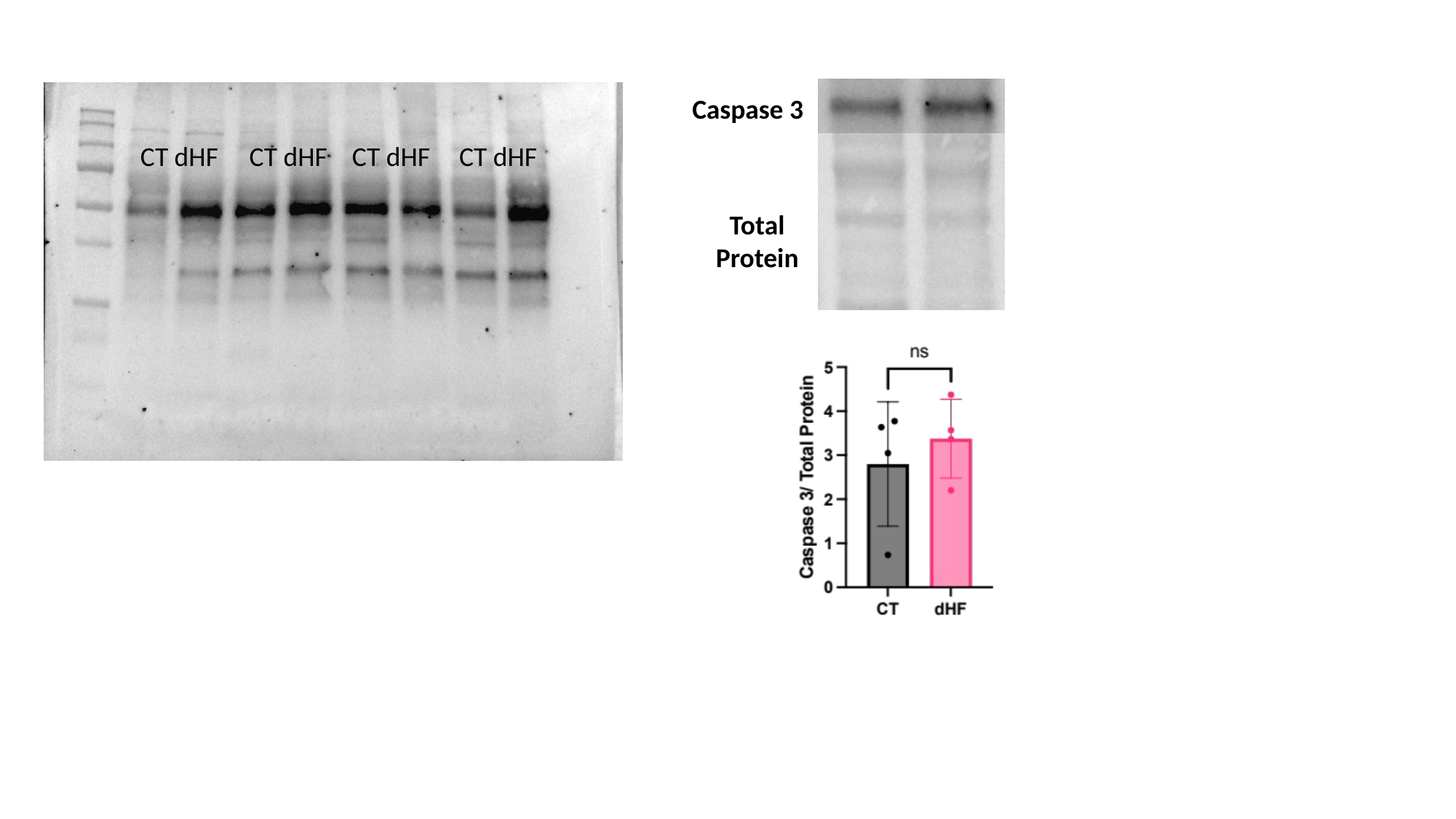

CT dHF
CT dHF
CT dHF
CT dHF
Caspase 3
Total Protein

## Slide 15
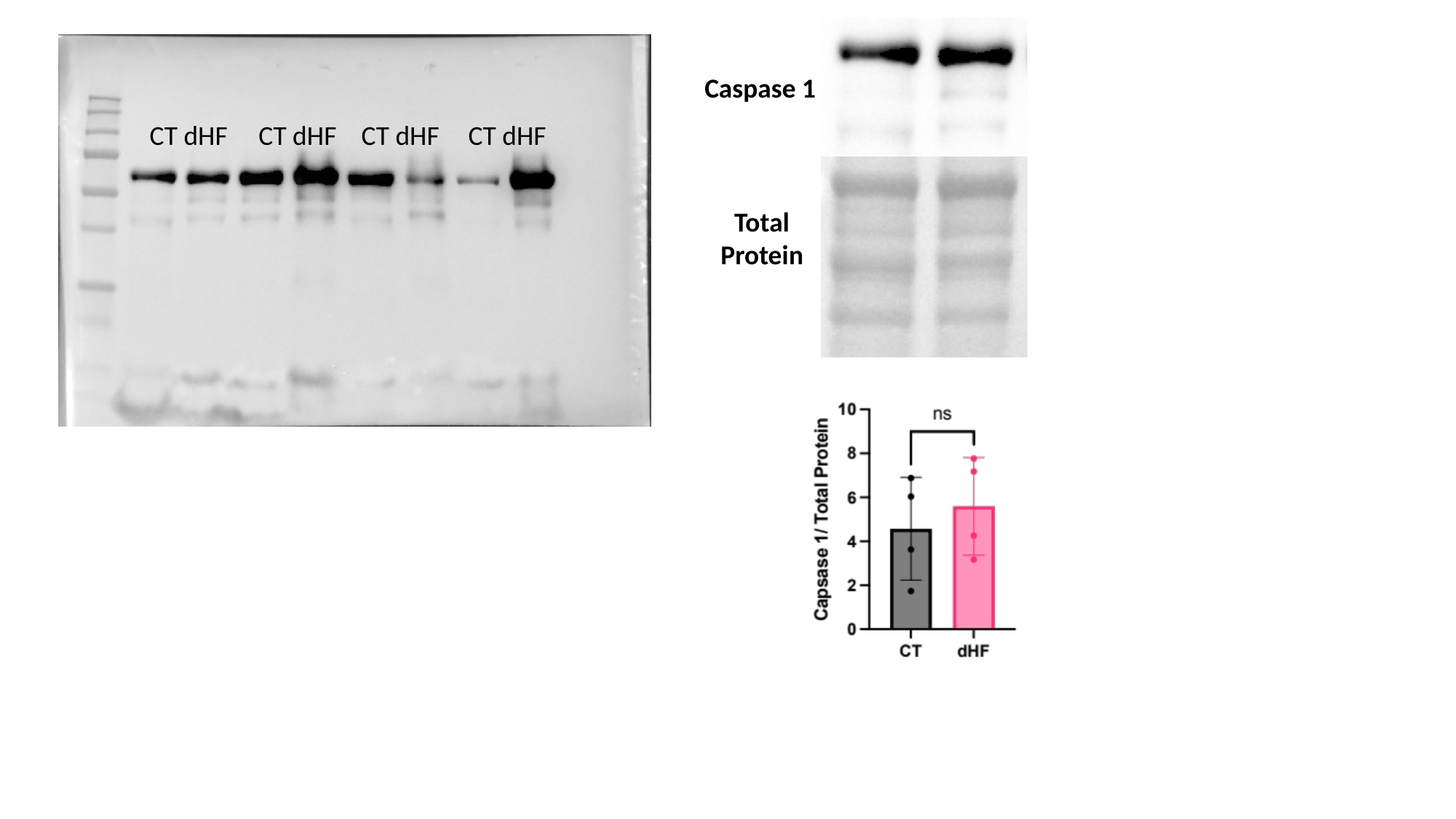

CT dHF
CT dHF
CT dHF
CT dHF
Caspase 1
Total Protein

## Slide 16
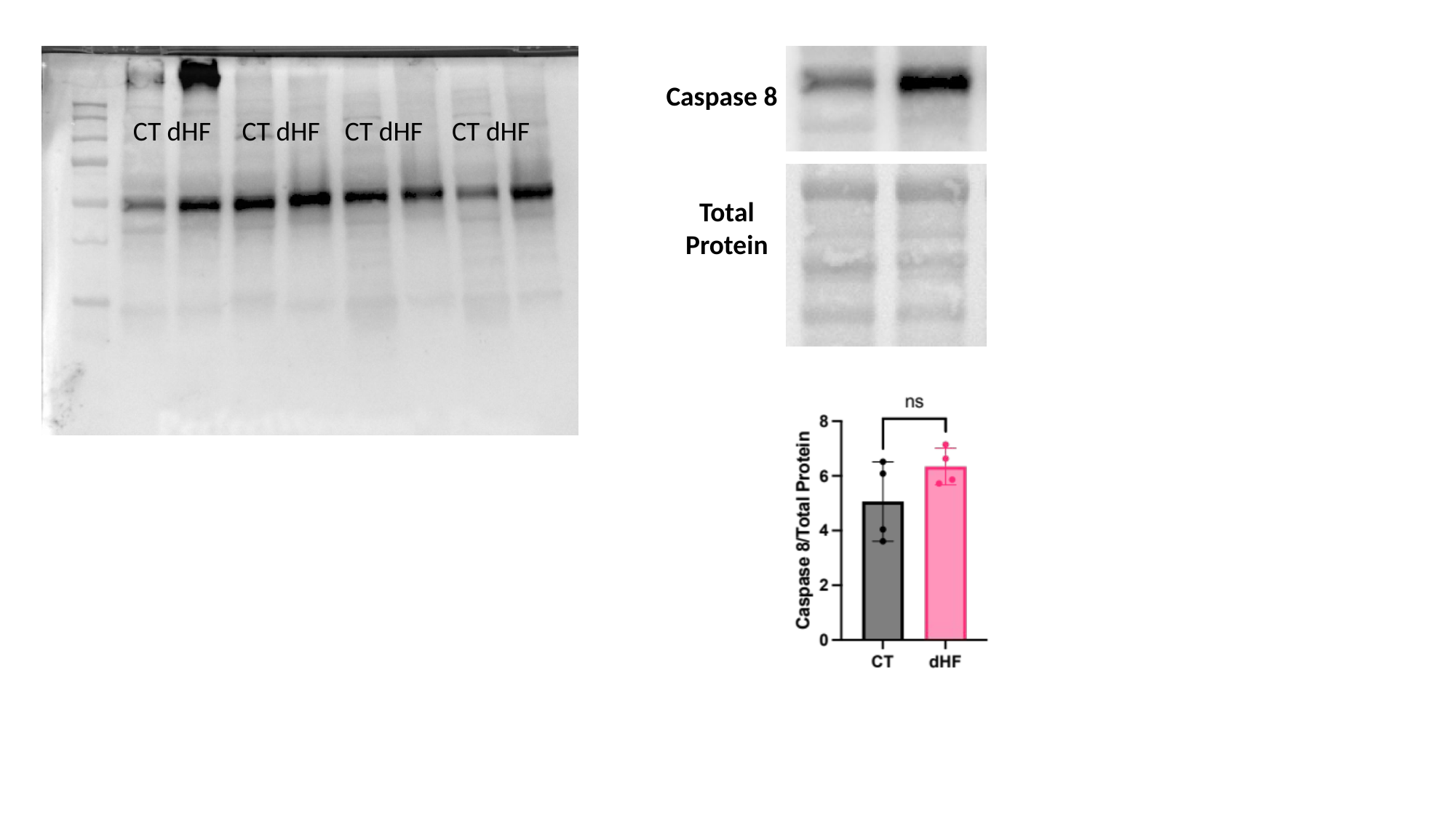

CT dHF
CT dHF
CT dHF
CT dHF
Caspase 8
Total Protein

## Slide 17
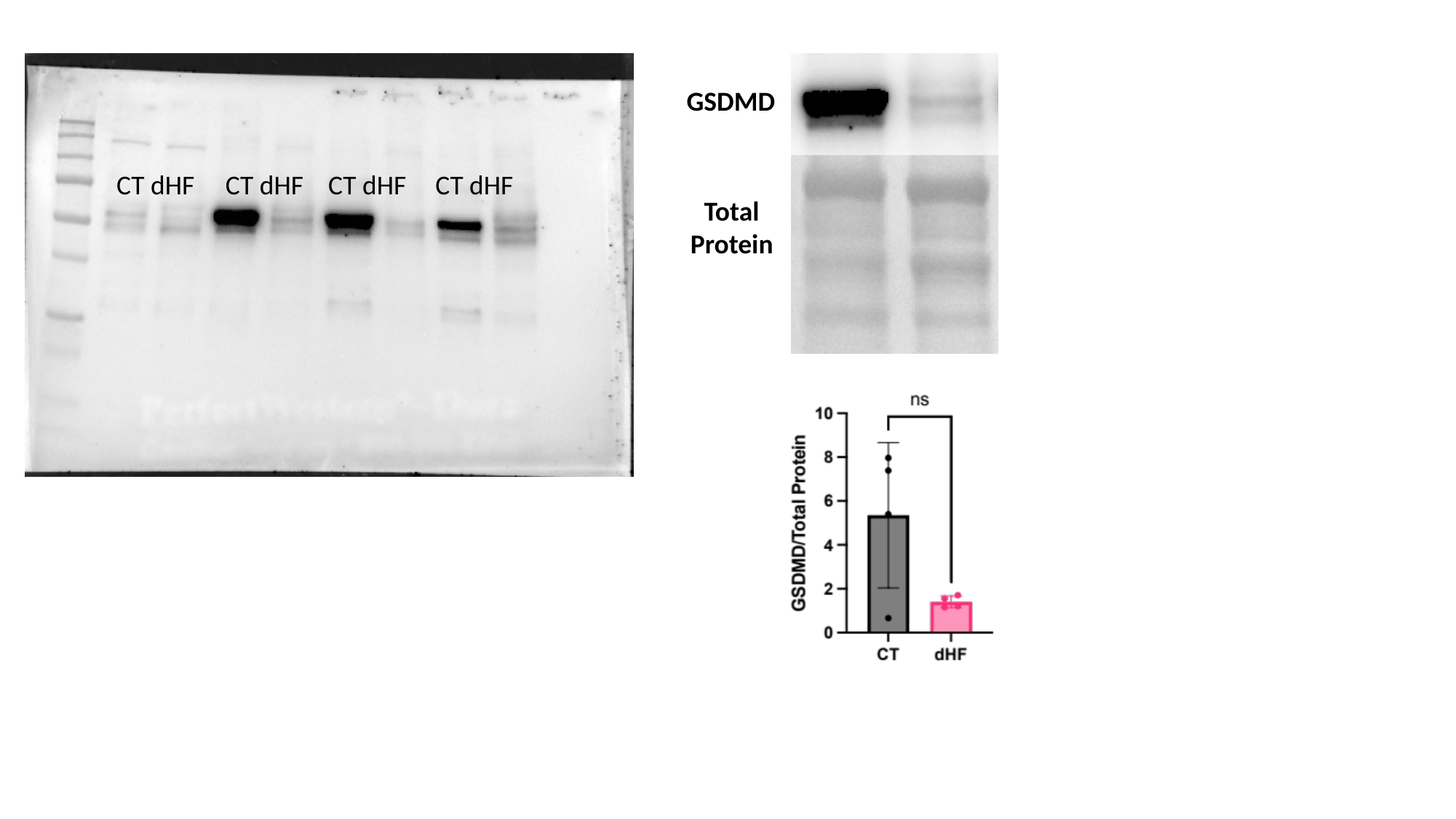

CT dHF
CT dHF
CT dHF
CT dHF
GSDMD
Total Protein

## Slide 18
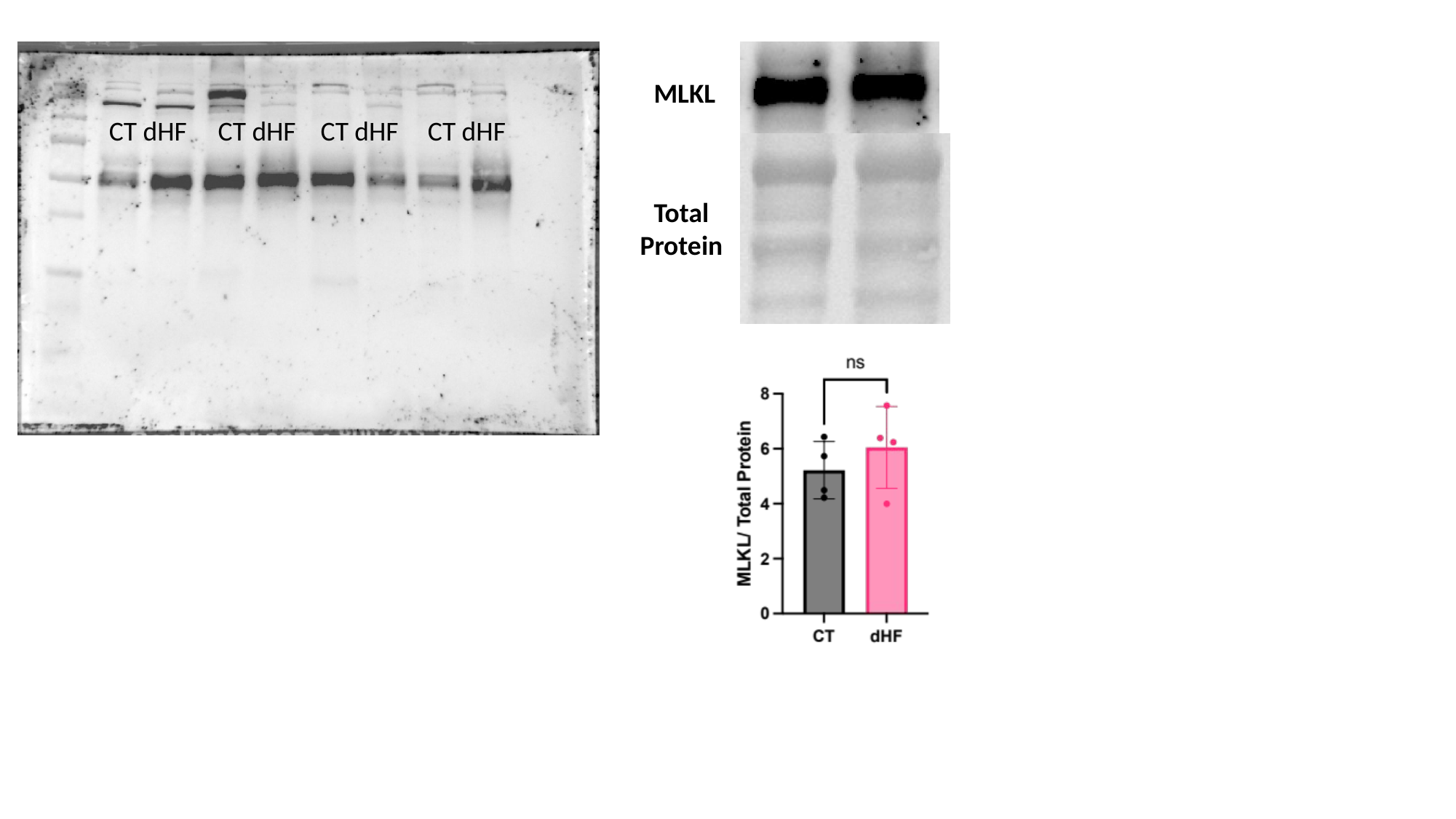

CT dHF
CT dHF
CT dHF
CT dHF
MLKL
Total Protein
